# An updated meta-analysis of Chinese herbal medicine for the prevention of COVID-19 based on Western-Eastern medicine

**DOI:** 10.3389/fphar.2023.1257345

**Published:** 2023-11-13

**Authors:** Siying Hu, Dan Luo, Qikui Zhu, Jie Pan, Bonan Chen, Michael Furian, Harsh Vivek Harkare, Shoukai Sun, Adel Fansa, Xiaoping Wu, Baili Yu, Tianhong Ma, Fei Wang, Shihua Shi

**Affiliations:** ^1^ Chengdu University of Traditional Chinese Medicine, Chengdu, China; ^2^ Department of Biomedical Engineering and Computer and Data Science, Case Western Reserve University, Cleveland, OH, United States; ^3^ Department of Pathology, Stanford University School of Medicine, Palo Alto, CA, United States; ^4^ Department of Anatomical and Cellular Pathology, State Key Laboratory of Translational Oncology, Prince of Wales Hospital, The Chinese University of Hong Kong, Hong Kong, Hong Kong SAR, China; ^5^ Research Department, Swiss University of Traditional Chinese Medicine, Bad Zurzach, Switzerland; ^6^ Department of Epidemiology and Public Health, Swiss Tropical and Public Health Institute, Basel, Switzerland; ^7^ Faculty of Science, University of Basel, Basel, Switzerland; ^8^ Charité Universitätsmedizin Berlin, Berlin, Germany; ^9^ Faculty of Medicine, University of Basel, Basel, Switzerland; ^10^ National Clinical Research Center for Chinese Medicine Cardiology, Xiyuan Hospital, China Academy of Chinese Medical Sciences, Beijing, China; ^11^ Hospital of Chengdu University of Traditional Chinese Medicine, Chengdu, China; ^12^ Friedrich Miescher Institute for Biomedical Research, Basel, Switzerland

**Keywords:** Chinese herbal medicine, COVID-19, prevention, immunity, meta-analysis, WE medicine

## Abstract

**Background and aims:** Chinese herbal medicine (CHM) was used to prevent and treat coronavirus disease 2019 (COVID-19) in clinical practices. Many studies have demonstrated that the combination of CHM and Western medicine can be more effective in treating COVID-19 compared to Western medicine alone. However, evidence-based studies on the prevention in undiagnosed or suspected cases remain scarce. This systematic review and meta-analysis aimed to investigate the effectiveness of CHM in preventing recurrent, new, or suspected COVID-19 diseases.

**Methods:** We conducted a comprehensive search using ten databases including articles published between December 2019 and September 2023. This search aimed to identify studies investigating the use of CHM to prevent COVID-19. Heterogeneity was assessed by a random-effects model. The relative risk (RR) and mean differences were calculated using 95% confidence intervals (CI). The modified Jadad Scale and the Newcastle-Ottawa Scale (NOS) were employed to evaluate the quality of randomized controlled trials and cohort studies, respectively.

**Results:** Seventeen studies with a total of 47,351 patients were included. Results revealed that CHM significantly reduced the incidence of COVID-19 (RR = 0.24, 95% CI = 0.11–0.53, *p* = 0.0004), influenza (RR = 0.37, 95% CI = 0.18–0.76, *p* = 0.007), and severe pneumonia exacerbation rate (RR = 0.17, 95% CI = 0.05–0.64, *p* = 0.009) compared to non-treatment or conventional control group. Evidence evaluation indicated moderate quality evidence for COVID-19 incidence and serum complement components C3 and C4 in randomized controlled trials. For the incidence of influenza and severe pneumonia in RCTs as well as the ratio of CD4^+^/CD8^+^ lymphocytes, the evidence quality was low. The remaining outcomes including the disappearance rate of symptoms and adverse reactions were deemed to be of very low quality.

**Conclusion:** CHM presents a promising therapeutic option for the prevention of COVID-19. However, additional high-quality clinical trials are needed to further strengthen evidential integrity.

## 1 Introduction

Since the initial outbreak in late 2019, COVID-19 has evolved from an isolated incident to a global pandemic, affecting the whole world with very high transmission risk levels (World Health Organization, 2020). Multiple infectious disease models have indicated that the average basic reproduction number (R0) value of COVID-19 is approximately 3.28, surpassing the R0 value (2.9) of the severe acute respiratory syndrome (SARS) (Liu Y. et al., 2020). Importantly, research has demonstrated that all population groups are susceptible to the severe acute respiratory syndrome coronavirus 2 (SARS-CoV-2) with no discernible variation in susceptibility among different population groups (Cheng et al., 2020; Ogando et al., 2020; Stokes et al., 2020). Although the COVID-19 outbreak is no longer classified as a public health emergency, nearly 1.4 million new cases and over 1,800 deaths have been reported between 31 July and 27 August 2023 (World Health Organization, 2023a). As of 27 August 2023, the total number of confirmed cases worldwide has exceeded 770 million, resulting in over 6.9 million deaths (World Health Organization, 2023b). In light of the global spread of SARS-CoV-2, effective prevention and treatment remain a focal concern. Several antiviral drugs, including Remdesivir, Paxlovid, and Molnupiravir, and monoclonal antibody drugs, such as casirivimab + imdevimab, were developed during the epidemic. However, these drugs do not fully meet clinical needs due to the continuous emergence of new variants of SARS-CoV-2, as well as the increasing recurrent or suspected COVID-19 relevant diseases (Combes et al., 2022; Ou et al., 2022). While vaccines have been deployed to prevent COVID-19 (Mohd et al., 2021), some variants exhibit reduced sensitivity to vaccine-induced neutralizing antibodies, and vaccinations cannot provide complete protection from the disease due to the lengthy development cycle, lagging impact, and high viral variability (Sultana et al., 2020; Sgorlon et al., 2023). An increased risk of myocarditis and pericarditis was identified along with an increased incidence of cardiac death following vaccinations (Diaz et al., 2021; Oster et al., 2022; Nafilyan et al., 2023). For specific populations, such as immunocompromised individuals or those with a history of targeted drug therapy, novel coronavirus vaccines may fail to activate adequate immune responses, making it difficult to achieve a satisfactory preventive effect even with an increased dose (Shoham et al., 2023; Calabrese et al., 2023). Currently, all these aforementioned factors present new challenges.

For most suspected cases nowadays, individuals may not recognize the importance of treatment and of seeking nucleic acid amplification testing. However, even after the results of nucleic acid tests turn negative after a SARS-CoV-2 infection, there remains a chance that clinical symptoms persist and new symptoms gradually appear, a condition known as Long COVID. Various clinical symptoms of Long COVID including cough, shortness of breath, headache, insomnia, anxiety, palpitations, and anosmia, continuously disturb the quality of life (Zadeh et al., 2023). Current Long COVID treatment regimens are based on small pilot studies and still need further validation of effectiveness from larger clinical studies (Davis et al., 2023), therefore their effectiveness is still under debate. Furthermore, the neutralizing antibodies produced during the initial infection decline over time and thus cannot effectively prevent the occurrence of subsequent infections, leading to common re-infections (Fang et al., 2023), which further increase the overall mortality rate and risk of Long COVID. Thus, there is an urgent need for measures to prevent re-infection (Boufidou et al., 2023). Due to the high contagiosity of COVID-19, increased risk of mortality rate from critical illness (Jia and Gong, 2021), and the emergence of associated sequelae (Samanta et al., 2022; Stefanou et al., 2022), preventative measures for recurrent, new, or suspected COVID-19 cases are warranted.

Western and Eastern medicine (referred to as “WE” medicine), a medical paradigm that integrates Western Medicine (focusing on microscopic and single disease targets) and Eastern Medicine (such as traditional Chinese medicine, focusing on holism and treatments based on syndrome differences) (Belli., 2020; Luo et al., 2023), could offer a breakthrough in addressing the complex COVID-19 challenge on suspected, recurrent, or new cases. CHM has a unique history in the prevention and treatment of pandemics. From ancient plagues to SARS and the current COVID-19 pandemic, CHM has played an important role (Wu et al., 2021). Previous published systematic analyses confirm that early intervention with CHM for COVID-19 is effective in reducing mortality, lowering the incidence of severe cases, and improving clinical symptoms (Kang et al., 2022; Xu et al., 2022; Zhuang et al., 2022). However, evidence-based studies on the prevention of COVID-19 with suspected or recurrent cases remain scarce. Growing evidence suggests that CHM, as an adjuvant intervention, can assist in boosting the immune system and reducing clinical symptoms more effectively than conventional treatment alone (An et al., 2021; Huang et al., 2021; Ren et al., 2020). Given the lack of conclusive evidence on the effectiveness of CHM on COVID-19 prevention, this study aimed to investigate whether CHM could prevent new, recurrent, or suspected cases of COVID-19.

## 2 Methods

### 2.1 Protocol and registration

This study was registered in the PROSPERO database (CRD42021231297) and conducted following the standards of the Preferred Reporting Items for Systematic Reviews and Meta-Analyses (PRISMA) (Page et al., 2021).

### 2.2 Search strategies

We conducted searches across ten electronic databases, including PubMed, the Cochrane Library, Science Direct, Google Scholar, Embase, the Web of Science, China National Knowledge Infrastructure Database, Wanfang Database, Chongqing VIP Chinese Science and Technology Periodical, and SinoMed.

Studies published from December 2019 to September 2023 were considered without restrictions on languages or regions to reduce publication bias. We also searched the WHO COVID-19 website (World Health Organization, 2023c). Moreover, to minimize the risk of missing relevant studies, we performed manual searches based on references from identified studies and reviews.

Search terms related to COVID-19, Traditional Chinese Medicine, and clinical trials were used. The search strategy was made based on the requirements of different databases. Detailed search strategies are provided in the attachment.

### 2.3 Selection criteria

The titles, abstracts, and full texts of the retrieved articles were independently screened, extracted, and cross-checked by two researchers (SH and DL). Any disagreements were discussed by the two researchers, and if not resolved, a final decision was taken by a third reviewer (FW). Table 1 presents the Population, Intervention, Comparison, Outcomes, and Study (PICOS) design.

**TABLE 1:** PICOS for study selection.

Parameters	Descriptions
**Patients**	Uninfected individuals (including recovered cases) and suspected cases of COVID-19
**Intervention**	Chinese herbal medicine plus conventional pharmaceutical treatment, or Chinese herbal medicine used alone
**Comparison**	Conventional pharmaceutical treatment, or no treatments
**Outcome**	COVID-19 incidence; influenza incidence; severe pneumonia incidence; IgA; IgM; IgG; C3; C4; CD4^+^/CD8^+^; disappearance rate of fever; disappearance rate of cough; disappearance rate of sputum; disappearance rate of nasal obstruction; disappearance rate of runny nose; disappearance rate of sore throat; disappearance rate of shortness of breath; disappearance rate of fatigue; disappearance rate of muscle pain; disappearance rate of poor appetite; adverse reaction
**Setting**	Randomized controlled trials (If not available, observational studies)

The inclusion criteria were as follows: 1) Participants were uninfected individuals (including recovered cases) and suspected cases of COVID-19. Suspected cases were defined as patients with an epidemiological history or any new-onset fever and respiratory symptoms (World Health Organization, 2020; The General Office of the National Health Commission and the Office of the State Administration of Traditional Chinese Medicine, 2022); 2) A blank control group was designed, or participants in the control group were treated with conventional Western medicine (anti-virus, anti-infection, adjuvant supportive therapy, *etc.*) or placebo. Participants in the treatment group were treated with CHM (such as CHM patent medicine, CHM decoction, CHM granules, CHM oral liquids, *etc.*); 3) At least one outcome measure was reported, such as COVID-19 incidence, influenza incidence, severe pneumonia incidence, immunoglobulin (Ig)A, IgM, IgG, serum complement (C)3, C4, CD4^+^/CD8^+^ ratio, disappearance rate of fever, disappearance rate of cough, disappearance rate of sputum, and disappearance rate of nasal obstruction; 4) An acceptable study design was included: randomized controlled trials (RCTs), quasi-randomized controlled trials, cohort studies, and case-control studies.

The exclusion criteria were as follows: 1) Studies, that initially recruited COVID-19 patients; 2) conference reports, case reports, reviews, and pharmacological research; 3) Studies without quantitative data; 4) Studies without the description of the type of research; 5) Studies without outcome measures.

### 2.4 Outcomes

The primary outcomes included the incidence of COVID-19, influenza and severe pneumonia. The secondary outcomes were immunological parameters (IgA, IgM, IgG, C3, C4 and CD4^+^/CD8^+^), disappearance rate of symptoms (fever, cough, sputum, nasal obstruction, runny nose, sore throat, shortness of breath, fatigue, muscle pain and poor appetite), and adverse reactions.

### 2.5 Data extraction and management

Two researchers (QZ and JP) simultaneously and independently extracted participant characteristics, research processes, and outcome indicators. Extraction forms were crosschecked after completion. Disputes were resolved through discussion with a third investigator (SSh). If the outcome data were presented as the median and interquartile range, then the data were further calculated based on the sample size and the estimation methods reported in the relevant literature (Wan et al., 2014; Luo et al., 2018) to obtain standard deviation.

### 2.6 Critical appraisal

Methodological quality evaluation was separately conducted by two reviewers (BC and TM) and then double-checked. For RCTs, the modified Jadad scale was used for scoring. The modified Jadad scale scoring method was evaluated based on the random sequence of the literature, blinding, and whether dropouts and withdrawals were reported (Jadad et al., 1996). A total score between 4 and 7 suggests high-quality research, whereas a score between 1 and 3 indicates low-quality research. The Newcastle-Ottawa score (NOS) was used to evaluate the overall quality of cohort studies. Disputes were settled through discussion with a third investigator (FW).

### 2.7 Statistical analysis

The RevMan5.4.1 software (The Nordic Cochrane Center, 21 The Cochrane Collaboration, Copenhagen, Denmark) was used to perform the meta-analysis. Odds ratios (OR) and 95% CI were used for binary variables. Continuous variables were represented by either the weighted mean difference (WMD) or the standardized mean difference (SMD), depending on whether the measurement units of outcomes were the same. The effect size was calculated using a 95% CI. The *I* ^*2*^ test was applied to evaluate the heterogeneity of pooled studies. A fixed-effect model was utilized when there was no statistical heterogeneity among the included studies or when the statistical heterogeneity was minor (<50%). Otherwise, a random-effects model was used (Higgins et al., 2003). Subgroup and sensitivity analyses were conducted to resolve heterogeneity. Furthermore, GRADEpro (GradePro, Houston, TX, United States) was used to assess the quality of the evidence and the robustness of the recommendations for the outcome indicators. The quality of evidence was ranked as high, moderate, low, or very low, depending on its strength, considering five criteria including publication bias, risk of bias, inconsistency, indirectness, and imprecision.

## 3 Results

### 3.1 Study selection

**FIGURE 1:**
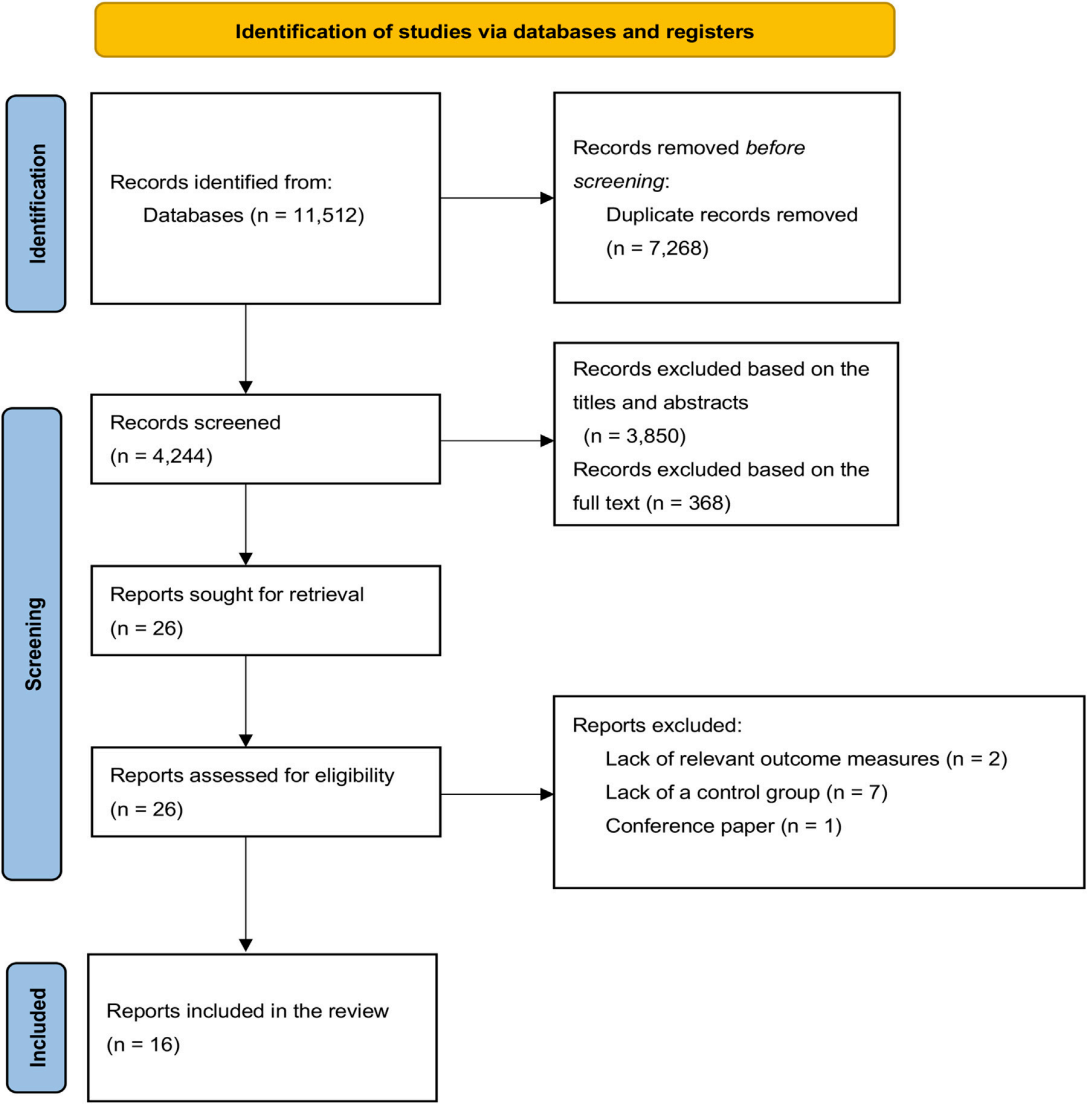
Flow diagram for the process of included studies.

Following PICOS principles, a total of 11,512 publications were initially identified, of which 7,268 were duplicates. 3,850 were excluded at the titles and abstracts screening level, and 368 articles were excluded after full-text evaluation. Accordingly, 19 papers were included in the systematic review. However, 16 publications (Fang et al., 2020; Liu et al., 2020b; Lv et al., 2020; Wang et al., 2020; Yan et al., 2021; Wang F et al., 2021; Wang YL et al., 2021; Wang ZZ et al., 2021; Xiao et al., 2021; Yang et al., 2021; Zhang, 2021; Zheng and Lu, 2021; Zheng, 2021; Wang et al., 2022; Liu et al., 2023; Xie et al., 2023) were included in the meta-analysis as two of those articles did not have any relevant outcome indicators, and one (Gong et al., 2022) was a conference paper. Figure 1 illustrates the literature screening and selection process.

### 3.2 Characteristics of the included studies

The selection process resulted in the inclusion of 16 articles, consisting of 12 RCTs and 5 cohort studies. 15 of these (Fang et al., 2020; Liu et al., 2020b; Lv et al., 2020; Wang et al., 2020; Wang F et al., 2021; Wang YL et al., 2021; Wang ZZ et al., 2021; Xiao et al., 2021; Yang et al., 2021; Zhang, 2021; Zheng and Lu, 2021; Zheng, 2021; Liu et al., 2023; Xie et al., 2023) were written in Chinese, and 2 studies (Wang et al., 2022; Yan et al., 2021) were written in English. The included studies collectively encompassed a total of 47,351 patients. Among these, one was a multi-center trial (Yan et al., 2021) while the remaining ones were single-centered studies (Fang et al., 2020; Liu et al., 2020b; Lv et al., 2020; Wang et al., 2022; Wang et al., 2020; Wang F et al., 2021; Wang YL et al., 2021; Wang ZZ et al., 2021; Xiao et al., 2021; Yang et al., 2021; Zhang, 2021; Zheng and Lu, 2021; Zheng, 2021; Liu et al., 2023; Xie et al., 2023). All the studies were conducted in China, spanning from January 2020 to July 2022.

In terms of interventions, the experimental groups primarily received Chinese patent medicine, Chinese medicine decoction, and Chinese medicine formula granules. In contrast, the control groups underwent symptomatic life support treatment or blank control. The fundamental aspects of the studies are provided in Table 2.

**TABLE 2:** Summary of clinical characteristics of included studies.

Study (author/year)	Location	Number of patients		Age (year)		Gender (male %)		Intervention		Treatment course	Admission	Population inclusion	Data Selected
		I	C	I	C	I	C	I	C		time		
Yan et al., (2021)	Chengdu, Sichuan Province	11092	10973	47.29 ± 16.72	51.67 ± 18.48	48.67	47.27	Huoxiang Zhengqi oral liquids (10mL, bid)	N	5d	2020.1.30–2020.2.29	Healthy people	1, 2, 20
								Jinhao Jiere granules (8g, bid)					
Wang ZZ et al. (2021)	Linxia, Gansu Province	927	927	NR	NR	31.39	31.39	Yupingfeng powder prescription (200mL, bid)	N	5-7d	2020.1.28–2.29	Healthy people	1, 2, 20
Zheng. (2021)	Taian, Shandong Province	240	240	NR	NR	NR	NR	Yupingfeng powder prescription (1 dose/d, 150mL, bid)	N	14d	2020.1–2020.4	Healthy people	1, 3, 20
Wang YL et al. (2021)	Zhuhai, Guangdong Province	5128	5128	40.43 ± 11.84	42.36 ± 12.96	42.18	48.38	Xiao chaihu decoction and Yupingfeng powder prescription (1 bag, bid)	N	7d	2020.2.20–2021.1.20	Healthy people	1, 2
Zheng. (2021)	Fuzhou, Fujian Province	30	30	43.56 ± 8.87	43.86 ± 9.1	56.67	46.67	CHM (external use, qd)	N	30d	2020.2–2020.11	Healthy people	4, 5, 6
Xiao et al. (2021)	Zhuzhou, Hunan Province	50	50	58.37 ± 5.28	59.16 ± 5.34	68	66	COVID-19 Prevention Prescription No. 2 (1 dose/d, 200mL, bid) + C	cpt	63d	2020.1.1–2020.3.15	Patients with lung cancer after chemotherapy	9
Liu et al. (2020a)	Zhuzhou, Hunan Province	151	51	45.83 ± 3.40	45.43 ± 4.33	37.08	35.29	COVID-19 Prevention Prescription No. 1 (1 dose/d, 200mL, bid)	N	4d	2020.3.15–2020.4.7	Healthy young people	4, 5, 6, 7, 8, 9
Liu et al. (2020b)	Zhuzhou, Hunan Province	146	50	65.35 ± 5.24	65.35 ± 7.63	35.61	40	COVID-19 Prevention Prescription No. 1 (1 dose/d, 200mL, bid)	N	4d	2020.3.15–2020.4.7	Healthy older people	4, 5, 6, 7, 8, 9
Wang F et al. (2021)	Mianyang, Sichuan Province	494	590	NR	NR	32.39	28.47	Yiqi Fanggan recipe (2 dose/3 d, 200mL, bid)	N	7d	2020.5–2020.7	Healthy people	1, 2
Yang et al. (2021)	Shenzhen, Guangdong Province	17	17	45.6 ± 12.5	47.7 ± 14.6	35.29	47.06	Jiegeng Xingren flavored formula (1 dose/d, 200mL, bid) + C	cpt	14d	2020.1.28–2020.2.28	Suspected COVID-19	10, 11, 17, 19
Zheng and Lu. (2021)	Shenyang, Liaoning Province	18	14	51.2 ± 19.4	53.1 ± 20.0	55.56	50	Xiao chaihu decoction and Yupingfeng powder prescription (1 dose/d, 100mL, bid) + C	cpt	5d	2020.1.25–2020.3.14	Suspected COVID-19	10, 11, 12, 15, 19
Wang et al. (2020)	Shenzhen, Guangdong Province	90	90	37.23 ± 12.48	36.65 ± 13.57	67.78	68.89	Suspected COVID-19 formula No. 1 (200mL, bid) + C	cpt	3d	2020.2.7–2020.3.20	Suspected COVID-19	3
Lv et al. (2020)	Wuhan, Hubei Province	63	38	59.12 ± 16.56	60.20 ± 17.01	44.4	47.4	Lianhua Qingwen granules (1 bag, tid) + C	cpt	10d	2020.1.1–2020.1.27	Suspected COVID-19	10, 11, 12, 13, 14, 15, 16, 17, 18, 19, 20
Fang et al. (2020)	Xiangyang, Hubei Province	42	41	4.8 ± 3.7	3.9 ± 3.2	42.86	39.02	Lianhua Qingwen granules + C	cpt	5d	2020.1.28–2020.3.31	Suspected COVID-19	10, 11, 12, 13, 14, 16, 18
Wang et al. (2022)	Yangzhou, Jiangsu Province	1016	270	41.0 (Median)	35.0 (Median)	46.77	50	Qingfei Paidu Decoction and Fuzhengyiqin-g prescription (1 dose/2 d, 100mL, bid)	N	6d	2020.8.4–2020.9.5	Suspected COVID-19	1
Liu et al. (2023)	Shanghai	4385	4821	50.00 (34.00, 54.50) (IQR)	47.00 (31.50, 54.00) (IQR)	68.27	66.07	Qiangshen Kangyi Decoction (1 dose/d, bid)	N	7d	2022.4–2022.7	Healthy people	1, 20
Xie et al. (2023)	Xi’an, Shanxi Province	66	66	31.86 ± 6.75	29.71 ± 6.53	18.19	24.24	Yiqi Kangfei decoction (1 dose/d, 200mL, bid)	N	14d	2021.12–2022.3	Suspected COVID-19	1, 20

C, control group; I, intervention group; NR, not reported in original studies; CPT, conventional pharmaceutical treatment (the same as drugs in comparison group); N, no treatment.

Outcomes: 1, COVID-19, incidence; 2, influenza incidence; 3, severe pneumonia incidence; 4, IgA; 5, IgM; 6, IgG; 7, C3; 8, C4; 9, CD4^+^/CD8^+^; 10, disappearance rate of fever; 11, disappearance rate of cough; 12, disappearance rate of sputum; 13, disappearance rate of nasal obstruction; 14,disappearance rate of runny nose; 15, disappearance rate of sore throat; 16,disappearance rate of shortness of breath; 17,disappearance rate of fatigue; 18, disappearance rate of muscle pain; 19, disappearance rate of poor appetite; 20, adverse reactions.

### 3.3 Description of CHM

This review included 15 traditional Chinese herbal formulas, all well-known recipes with long history in China. These formulas include Huoxiang Zhengqi oral liquids, Jinhao Jiere granules, Yupingfeng powder prescription, Moxibustion, COVID-19 Prevention Prescription No. 1, COVID-19 Prevention Prescription No. 2, Yiqi Fanggan recipe, Jiegeng Xingren flavored formula, Xiao chaihu decoction, Formula no. 1 for suspected COVID-19, Lianhua Qingwen granules, Qingfei Paidu Decoction, Fuzhengyiqi prescription, Qiangshen Kangyi Decoction and Yiqi Kangfei decoction. The preparations employed included decoction, oral liquids, granules, and moxibustion. Additionally, analysis of the distinctive flavors of each Chinese herbal formula depicted a total of 77 different types of Chinese botanical drugs, of which seven were the most popular, including - as shown in “scientific plant name” [family; synonyms](Heinrich et al., 2022): Astragalus mongholicus Bunge [Fabaceae; Astragali R.] (66.67%), Atractylodes macrocephala Koidz. [Asteraceae; Atractylis macrocephala (Koidz.) Hand. -Mazz.] (53.33%), Saposhnikovia divaricata (Turcz. ex Ledeb.) Schischk. [Apiaceae; Ledebouriella seseloides (Hoffm.) H. Wolff] (53.33%), Glycyrrhiza uralensis Fisch. ex DC. [Fabaceae; Glycyrrhiza asperrima var. uralensis (Fisch. ex DC.) Regel & Herder] (73.33%), Agastache rugosa (Fisch. & C.A.Mey.) Kuntze [Lamiaceae; Agastache formosana (Hayata) Hayata ex Makino & Nemoto] (80.00%), *Lonicera japonica* Thunb. [Caprifoliaceae; Caprifolium japonicum (Thunb.) Dum. Cours.] (53.33%), and Forsythia suspensa (Thunb.) Vahl [Oleaceae Syringa suspensa Thunb.] (46.67%). The composition and characteristics of CHM were listed in Table 3.

**TABLE 3:** Description of CHM.

References	Name of CHM (type of formula)	Source	Extraction process	Composition of CHM (g/day)
Yan et al. (2021)	Huoxiang Zhengqi oral liquids (oral liquids)	Jinhao Jiere granules (Taiji Group Co., Ltd. National Drug Approval Number B20020411; Batch numbers 18070013, 18070014, 18070015, 18070016; 8 g per bag)	Partially reported^a^	Jinhao Jiere granules: Artemisia annua L. [Asteraceae; Artemisia annua f. macrocephala Pamp.], *Lonicera japonica* Thunb. [Caprifoliaceae; Caprifolium japonicum (Thunb.) Dum.Cours.], Nepeta tenuifolia Benth. [Lamiaceae; Schizonepeta tenuifolia (Benth.) Briq.], Scutellaria baicalensis Georgi [Lamiaceae; Scutellaria macrantha Fisch. ex Rchb.], Saposhnikovia divaricata (Turcz. ex Ledeb.) Schischk. [Apiaceae; Ledebouriella seseloides (Hoffm.) H.Wolff], Platycodon grandiflorus (Jacq.) A. DC. [Campanulaceae; Campanula glauca Thunb.]. (One bag per time, twice daily)
	Jinhao Jiere granules (granules)	Huoxiang Zhengqi oral liquids (Taiji Group Co., Ltd. National Drug Approval Number Z50020409; Batch numbers 17110445, 17110446, 17110447; 10 mL per vial)		Huoxiang Zhengqi oral liquids: Atractylodes lancea (Thunb.) DC. [Asteraceae; Atractylis chinensis (Bunge) DC.], Citrus × aurantium f. deliciosa (Ten.) M.Hiroe [Rutaceae; Citrus × reticulata Blanco], Magnolia officinalis Rehder & E.H.Wilson [Magnoliaceae; Houpoea officinalis (Rehder & E.H.Wilson) N.H.Xia & C.Y.Wu], Angelica dahurica (Hoffm.) Benth. & Hook.f. ex Franch. & Sav. [Apiaceae; Callisace dahurica Hoffm.], Poria cocos (Schw.) Wolf [Polyporaceae], Areca catechu L. [Arecaceae; Areca catechu var. batanensis Becc.], Pinellia ternata (Thunb.) Makino [Araceae; Arum ternatum Thunb.], Glycyrrhiza uralensis Fisch. ex DC. [Fabaceae; Glycyrrhiza asperrima var. uralensis (Fisch. ex DC.) Regel & Herder], Agastache rugosa (Fisch. & C.A.Mey.) Kuntze [Lamiaceae; Agastache formosana (Hayata) Hayata ex Makino & Nemoto] oil, Perilla frutescens (L.) Britton [Lamiaceae; Ocimum frutescens L.] oil. (One vial per time, twice daily)
Liu et al. (2020a)	COVID-19 Prevention Prescription No. 1 (decoction)	The First Hospital Affiliated to Hunan Higher Vocational Colleges of Traditional Chinese Medicine	Partially reported^b^	Astragalus mongholicus Bunge [Fabaceae; Astragali R.] (15), Atractylodes macrocephala Koidz. [Asteraceae; Atractylis macrocephala (Koidz.) Hand. -Mazz.] (9), Forsythia suspensa (Thunb.) Vahl [Oleaceae; Syringa suspensa Thunb.] (9), Lonicera hypoglauca Miq. [Caprifoliaceae; Lonicera affinis var. hypoglauca (Miq.) Rehder] (9), Agastache rugosa (Fisch. & C.A. Mey.) Kuntze [Lamiaceae; Agastache formosana (Hayata) Hayata ex Makino & Nemoto] (6), Acorus calamus var. angustatus Besser [Acoraceae; Acorus tatarinowii Schott] (6), Saposhnikovia divaricata (Turcz. ex Ledeb.) Schischk. [Apiaceae; Ledebouriella seseloides (Hoffm.) H. Wolff] (6), Glycyrrhiza uralensis Fisch. ex DC. [Fabaceae; Glycyrrhiza asperrima var. uralensis (Fisch. ex DC.) Regel & Herder] (6).
Liu et al. (2020b)	COVID-19 Prevention Prescription No. 1 (decoction)	The First Hospital Affiliated to Hunan Higher Vocational Colleges of Traditional Chinese Medicine	Partially reported^b^	Astragalus mongholicus Bunge [Fabaceae; Astragali R.] (15), Atractylodes macrocephala Koidz. [Asteraceae; Atractylis macrocephala (Koidz.) Hand. -Mazz.] (9), Forsythia suspensa (Thunb.) Vahl [Oleaceae; Syringa suspensa Thunb.] (9), Lonicera hypoglauca Miq. [Caprifoliaceae; Lonicera affinis var. hypoglauca (Miq.) Rehder] (9), Agastache rugosa (Fisch. & C.A. Mey.) Kuntze [Lamiaceae; Agastache formosana (Hayata) Hayata ex Makino & Nemoto] (6), Acorus calamus var. angustatus Besser [Acoraceae; Acorus tatarinowii Schott] (6), Saposhnikovia divaricata (Turcz. ex Ledeb.) Schischk. [Apiaceae; Ledebouriella seseloides (Hoffm.) H. Wolff] (6), Glycyrrhiza uralensis Fisch. ex DC. [Fabaceae; Glycyrrhiza asperrima var. uralensis (Fisch. ex DC.) Regel & Herder] (6).
Wang et al. (2020)	Suspected COVID-19 formula No. 1 (decoction^c^)	Guangdong Yifang Pharmaceutical Factory;	Partially reported^b^	Bupleurum chinense DC. [Apiaceae; Bupleurum octoradiatum Bunge] (30), Scutellaria baicalensis Georgi [Lamiaceae; Scutellaria macrantha Fisch. ex Rchb.] (15), Pinellia ternata (Thunb.) Makino [Araceae; Arum ternatum Thunb.] (9), Codonopsis pilosula (Franch.) Nannf. [Campanulaceae; Campanumoea pilosula Franch.] (30), Gypsum Fibrosuum (30), Agastache rugosa (Fisch. & C.A. Mey.) Kuntze [Lamiaceae; Agastache formosana (Hayata) Hayata ex Makino & Nemoto] (15), Neolitsea cassia (L.) Kosterm. [Lauraceae; Cinnamomum cassia (L.) J. Presl] (15), Paeonia lactiflora Pall. [Paeoniaceae; Paeonia albiflora var. trichocarpa Bunge] (15), Cyrtomium fortunei J.Sm. [Polypodiaceae; Aspidium falcatum var. fortunei (J.Sm.) Nichols ex Makino] (15), Ziziphus jujuba Mill. [Rhamnaceae; Ziziphus sativa Gaertn. Fruct. Sem] (10), Zingiber officinale Roscoe [Zingiberaceae; Amomum zingiber L.] (6), Glycyrrhiza uralensis Fisch. ex DC. [Fabaceae; Glycyrrhiza asperrima var. uralensis (Fisch. ex DC.) Regel & Herder] (10). If accompanied by cough and little phlegm, add Magnolia officinalis Rehder & E.H. Wilson [Magnoliaceae; Houpoea officinalis (Rehder & E.H.Wilson) N.H.Xia & C.Y.Wu] (15), Perilla frutescens (L.) Britton [Lamiaceae; Ocimum frutescens L.] (15), Poria cocos (Schw.) Wolf [Polyporaceae] (30), Ophiopogon japonicus (Thunb.) Ker Gawl. [Asparagaceae; Convallaria japonica Thunb.] (30). If cough and with much phlegm, add Phragmites australis subsp. australis [Poaceae; Arundo phragmites L.] (30), Prunus persica (L.) Batsch [Rosaceae; Amygdalus persica L.] (10), Coix lacryma-jobi L. [Poaceae; Coix lacryma L.] (30), Benincasa hispida (Thunb.) Cogn. [Cucurbitaceae; Cucurbita hispida Thunb.] (30), Houttuynia cordata Thunb. [Saururaceae; Polypara cordata (Thunb.) H. Buek] (30). If the sore throat was noticeable, add Oroxylum indicum (L.) Kurz [Bignoniaceae; Bignonia indica L.] (10), Isatis tinctoria subsp. tinctoria [Brassicaceae; *Isatis indigotica* Fortune ex Lindl.] (30). If the headache was significant, add Hansenia weberbaueriana (Fedde ex H. Wolff) Pimenov & Kljuykov [Apiaceae; Notopterygium incisum K.C.Ting ex H.T.Chang] (10), Conioselinum anthriscoides ‘Chuanxiong’ [Apiaceae; Ligusticum chuanxiong] (30). If constipation, add Rheum palmatum L. [Polygonaceae; *Rhabarbarum palmatum* (L.) Moench] (10). If diarrhea significantly, add Poria cocos (Schw.) Wolf [Polyporaceae] (30), Atractylodes lancea (Thunb.) DC. [Asteraceae; Atractylis chinensis (Bunge) DC.] (10), Wurfbainia villosa (Lour.) Škorničk. & A.D.Poulsen [Zingiberaceae; Amomum villosum Lour.] (10).
		Shenzhen Hospital of Beijing University of Chinese Medicine (Longgang).		
Lv et al. (2020)	Lianhua Qingwen granules (granules)	Shijiazhuang Yiling Pharmaceutical Co., Ltd. Drug batch number 1812017; 6 g per bag.	NR	Forsythia suspensa (Thunb.) Vahl [Oleaceae; Syringa suspensa Thunb.], *Lonicera japonica* Thunb. [Caprifoliaceae; Caprifolium japonicum (Thunb.) Dum.Cours.], Ephedra sinica Stapf [Ephedraceae; Ephedra ma-huang Tang S.Liu], Prunus armeniaca var. armeniaca [Rosaceae; Armeniaca vulgaris var. ansu (Maxim.) T.T.Yu & L.T.Lu], Gypsum Fibrosuum, Isatis tinctoria subsp. tinctoria [Brassicaceae; *Isatis indigotica* Fortune ex Lindl.], Cyrtomium fortunei J.Sm. [Polypodiaceae; Aspidium falcatum var. fortunei (J.Sm.) Nichols ex Makino], Houttuynia cordata Thunb. [Saururaceae; Polypara cordata (Thunb.) H.Buek], Agastache rugosa (Fisch. & C.A.Mey.) Kuntze [Lamiaceae; Agastache formosana (Hayata) Hayata ex Makino & Nemoto], Rheum palmatum L. [Polygonaceae; *Rhabarbarum palmatum* (L.) Moench], Rhodiola rosea L. [Crassulaceae; Rhodiola sachalinensis Boriss.], Mentha canadensis L. [Lamiaceae; Mentha arvensis var. glabrata (Benth.)], Glycyrrhiza uralensis Fisch. ex DC. [Fabaceae; Glycyrrhiza asperrima var. uralensis (Fisch. ex DC.) Regel & Herder]. (One bag per time, 3 times daily)
Fang et al. (2020)	Lianhua Qingwen granules (granules)	Beijing Yiling Pharmaceutical Co., Ltd. Batch number: 1911009; 6 g per bag.	NR	Forsythia suspensa (Thunb.) Vahl [Oleaceae; Syringa suspensa Thunb.], *Lonicera japonica* Thunb. [Caprifoliaceae; Caprifolium japonicum (Thunb.) Dum.Cours.], Ephedra sinica Stapf [Ephedraceae; Ephedra ma-huang Tang S.Liu], Prunus armeniaca var. armeniaca [Rosaceae; Armeniaca vulgaris var. ansu (Maxim.) T.T.Yu & L.T.Lu], Gypsum Fibrosuum, Isatis tinctoria subsp. tinctoria [Brassicaceae; *Isatis indigotica* Fortune ex Lindl.], Cyrtomium fortunei J.Sm. [Polypodiaceae; Aspidium falcatum var. fortunei (J.Sm.) Nichols ex Makino], Houttuynia cordata Thunb. [Saururaceae; Polypara cordata (Thunb.) H.Buek], Agastache rugosa (Fisch. & C.A.Mey.) Kuntze [Lamiaceae; Agastache formosana (Hayata) Hayata ex Makino & Nemoto ], Rheum palmatum L. [Polygonaceae; *Rhabarbarum palmatum* (L.) Moench], Rhodiola rosea L. [Crassulaceae; Rhodiola sachalinensis Boriss.], Mentha canadensis L. [Lamiaceae; Mentha arvensis var. glabrata (Benth.)], Glycyrrhiza uralensis Fisch. ex DC. [Fabaceae; Glycyrrhiza asperrima var. uralensis (Fisch. ex DC.) Regel & Herder]. (2 g for Children ≤3 years old, 3 g for children aged 3–6 years, 6 g for children aged 6–14 years, 3 times daily)
Wang ZZ et al. (2021)	Yupingfeng powder prescription (decoction)	Linxia State Pharmaceutical Co.	Partially reported^b^	Astragalus mongholicus Bunge [Fabaceae; Astragali R.] (20), Atractylodes macrocephala Koidz. [Asteraceae; Atractylis macrocephala (Koidz.) Hand. -Mazz.] (15), Saposhnikovia divaricata (Turcz. ex Ledeb.) Schischk. [Apiaceae; Ledebouriella seseloides (Hoffm.) H.Wolff] (10), Hansenia weberbaueriana (Fedde ex H.Wolff) Pimenov & Kljuykov [Apiaceae; Notopterygium incisum K.C.Ting ex H.T.Chang] (6), Eupatorium fortunei Turcz. [Asteraceae; Eupatorium chinense var. tripartitum Miq.] (10), Zingiber officinale Roscoe [Zingiberaceae; Amomum zingiber L.] (6).
		Prepared by Linxia State People’s Hospital		
Zhang. (2021)	Fuzheng Gubiao Fanggan prescription (granlues)	NR	Partially reported^b^	Astragalus mongholicus Bunge [Fabaceae; Astragali R.] (12), Atractylodes macrocephala Koidz. [Asteraceae; Atractylis macrocephala (Koidz.) Hand. -Mazz.] (9),Saposhnikovia divaricata (Turcz. ex Ledeb.) Schischk. [Apiaceae; Ledebouriella seseloides (Hoffm.) H.Wolff] (6), Poria cocos (Schw.) Wolf [Polyporaceae] (12), Citrus × aurantium f. deliciosa (Ten.) M.Hiroe [Rutaceae; Citrus × reticulata Blanco] (6), Forsythia suspensa (Thunb.) Vahl [Oleaceae; Syringa suspensa Thunb.] (18), *Lonicera japonica* Thunb. [Caprifoliaceae; Caprifolium japonicum (Thunb.) Dum.Cours.] (10), Perilla frutescens (L.) Britton [Lamiaceae; Ocimum frutescens L.] (6), Glycyrrhiza uralensis Fisch. ex DC. [Fabaceae; Glycyrrhiza asperrima var. uralensis (Fisch. ex DC.) Regel & Herder] (3), Coix lacryma-jobi L. [Poaceae; Coix lacryma L.] (15), Agastache rugosa (Fisch. & C.A.Mey.) Kuntze [Lamiaceae; Agastache formosana (Hayata) Hayata ex Makino & Nemoto] (10), Platycodon grandiflorus (Jacq.) A. DC. [Campanulaceae; Campanula glauca Thunb.] (9).
Wang YL et al. (2021)	Fuzheng Gubiao granules (granules)	Zhuhai Integrated Hospital of Traditional Chinese and Western Medicine	Partially reported^a^	Astragalus mongholicus Bunge [Fabaceae; Astragali R.] (30), Bupleurum chinense DC. [Apiaceae; Bupleurum octoradiatum Bunge] (10), Pinellia ternata (Thunb.) Makino [Araceae; Arum ternatum Thunb.] (10), Codonopsis pilosula (Franch.) Nannf. [Campanulaceae; Campanumoea pilosula Franch.] (10), Ziziphus jujuba Mill. [Rhamnaceae; Ziziphus sativa Gaertn. Fruct. Sem] (10), Atractylodes macrocephala Koidz. [Asteraceae; Atractylis macrocephala (Koidz.) Hand. -Mazz. (10), Saposhnikovia divaricata (Turcz. ex Ledeb.) Schischk. [Apiaceae; Ledebouriella seseloides (Hoffm.) H.Wolff] (10), Agastache rugosa (Fisch. & C.A.Mey.) Kuntze [Lamiaceae; Agastache formosana (Hayata) Hayata ex Makino & Nemoto] (10), Eupatorium fortunei Turcz. [Asteraceae; Eupatorium chinense var. tripartitum Miq.] (10), Forsythia suspensa (Thunb.) Vahl [Oleaceae; Syringa suspensa Thunb.] (10), Scutellaria baicalensis Georgi [Lamiaceae; Scutellaria macrantha Fisch. ex Rchb.] (5), Glycyrrhiza uralensis Fisch. ex DC. [Fabaceae; Glycyrrhiza asperrima var. uralensis (Fisch. ex DC.) Regel & Herder] (5).
Xiao et al. (2021)	COVID-19 Prevention Prescription No. 2 (decoction)	The First Hospital Affiliated to Hunan Higher Vocational Colleges of Traditional Chinese Medicine	Partially reported^b^	Astragalus mongholicus Bunge [Fabaceae; Astragali R.] (30), *Lonicera japonica* Thunb. [Caprifoliaceae; Caprifolium japonicum (Thunb.) Dum.Cours.] (15), Citrus × aurantium f. deliciosa (Ten.) M.Hiroe [Rutaceae; Citrus × reticulata Blanco] (9), Ziziphus jujuba Mill. [Rhamnaceae; Ziziphus sativa Gaertn. Fruct. Sem] (5), Glycyrrhiza uralensis Fisch. ex DC. [Fabaceae; Glycyrrhiza asperrima var. uralensis (Fisch. ex DC.) Regel & Herder] (7).
Wang F et al. (2021)	Yiqi Fanggan recipe (decoction)	NR	Partially reported^d^	Astragalus mongholicus Bunge [Fabaceae; Astragali R.] (15), Atractylodes macrocephala Koidz. [Asteraceae; Atractylis macrocephala (Koidz.) Hand. -Mazz.] (10), Saposhnikovia divaricata (Turcz. ex Ledeb.) Schischk. [Apiaceae; Ledebouriella seseloides (Hoffm.) H.Wolff] (10), *Lonicera japonica* Thunb. [Caprifoliaceae; Caprifolium japonicum (Thunb.) Dum.Cours.] (10), Forsythia suspensa (Thunb.) Vahl [Oleaceae; Syringa suspensa Thunb.] (10), Agastache rugosa (Fisch. & C.A.Mey.) Kuntze [Lamiaceae; Agastache formosana (Hayata) Hayata ex Makino & Nemoto] (10), Atractylodes lancea (Thunb.) DC. [Asteraceae; Atractylis chinensis (Bunge) DC.] (10), Phragmites australis subsp. australis [Poaceae; Arundo phragmites L.] (10), Platycodon grandiflorus (Jacq.) A.DC. [Campanulaceae; Campanula glauca Thunb.] (10).
Yang et al. (2021)	Jiegeng Xingren flavored formula (granules)	Guangdong Fangfang Pharmaceutical Co., Ltd., prepared by the Traditional Chinese Medicine Department of Shenzhen Hospital of Southern Medical University.	Partially reported^b^	Platycodon grandiflorus (Jacq.) A. DC. [Campanulaceae; Campanula glauca Thunb.] (15), Prunus armeniaca var. armeniaca [Rosaceae; Armeniaca vulgaris var. ansu (Maxim.) T.T.Yu & L.T.Lu] (12), Forsythia suspensa (Thunb.) Vahl [Oleaceae; Syringa suspensa Thunb.] (10), Prunella vulgaris L. [Lamiaceae; Prunella vulgaris f. nana] (10), Fritillaria thunbergii Miq. [Liliaceae; Fritillaria verticillata var. thunbergii (Miq.) Baker] (10), Sargentodoxa cuneata (Oliv.) Rehder & E.H.Wilson [Lardizabalaceae; Holboellia cuneata Oliv.] (15), Paeonia × suffruticosa Andrews [Paeoniaceae; Paeonia × arborea C.C.Gmel.] (15), Phragmites australis subsp. australis [Poaceae; Arundo phragmites L.] (20), Glycyrrhiza uralensis Fisch. ex DC. [Fabaceae; Glycyrrhiza asperrima var. uralensis (Fisch. ex DC.) Regel & Herder] (6).
Zheng and Lu. (2021)	Xiao chaihu decoction and Yupingfeng powder prescription (decoction^c^)	Affiliated Hospital of Liaoning University of Traditional Chinese Medicine	Partially reported^b^	Bupleurum chinense DC. [Apiaceae; Bupleurum octoradiatum Bunge] (9), Scutellaria baicalensis Georgi [Lamiaceae; Scutellaria macrantha Fisch. ex Rchb.] (9), Pinellia ternata (Thunb.) Makino [Araceae; Arum ternatum Thunb.] (9), Cyrtomium fortunei J.Sm. [Polypodiaceae; Aspidium falcatum var. fortunei (J.Sm.) Nichols ex Makino] (9), *Lonicera japonica* Thunb. [Caprifoliaceae; Caprifolium japonicum (Thunb.) Dum.Cours.] (15), Forsythia suspensa (Thunb.) Vahl [Oleaceae; Syringa suspensa Thunb.] (15), Pseudostellaria heterophylla (Miq.) Pax [Caryophyllaceae; Pseudostellaria rhaphanorrhiza (Hemsl.) Pax] (10), Astragalus mongholicus Bunge [Fabaceae; Astragali R.] (15), Saposhnikovia divaricata (Turcz. ex Ledeb.) Schischk. [Apiaceae; Ledebouriella seseloides (Hoffm.) H.Wolff] (9), Atractylodes macrocephala Koidz. [Asteraceae; Atractylis macrocephala (Koidz.) Hand. -Mazz.] (9), Glycyrrhiza uralensis Fisch. ex DC. [Fabaceae; Glycyrrhiza asperrima var. uralensis (Fisch. ex DC.) Regel & Herder] (6). If the sore throat was severe, Arctium lappa L. [Asteraceae; Arctium lappa subsp. majus Arènes] (10), Platycodon grandiflorus (Jacq.) A. DC. [Campanulaceae; Campanula glauca Thunb.] (15), Taraxacum mongolicum Hand. -Mazz. [Asteraceae; *Taraxacum argute-denticulatum* Nakai & H.Koidz.] (10) were added. If had a cough, Platycodon grandiflorus (Jacq.) A. DC. [Campanulaceae; Campanula glauca Thunb.] (15), Prunus armeniaca var. armeniaca [Rosaceae; Armeniaca vulgaris var. ansu (Maxim.) T.T.Yu & L.T.Lu] (10), Magnolia officinalis Rehder & E.H.Wilson [Magnoliaceae; Houpoea officinalis (Rehder & E.H.Wilson) N.H.Xia & C.Y.Wu] (15) were added. If had a fever, Gypsum Fibrosuum (30), Anemarrhena asphodeloides Bunge [Asparagaceae; Terauchia anemarrhenifolia Nakai] (15) were added. If had diarrhea, Agastache rugosa (Fisch. & C.A.Mey.) Kuntze [Lamiaceae; Agastache formosana (Hayata) Hayata ex Makino & Nemoto] (10), Wurfbainia vera (Blackw.) Škorničk. & A.D.Poulsen [Zingiberaceae, Amomum krervanh Pierre ex Gagnep.] (10) were added.
Wang et al. (2022)	Qingfei Paidu Decoction (decoction); Fuzhengyiqing prescription (granules).	NR	Partially reported^b^	Qingfei Paidu Decoction: Ephedra sinica Stapf [Ephedraceae; Ephedra ma-huang Tang S.Liu] (4.5), Glycyrrhiza uralensis Fisch. ex DC. [Fabaceae; Glycyrrhiza asperrima var. uralensis (Fisch. ex DC.) Regel & Herder] (3), Prunus armeniaca var. armeniaca [Rosaceae; Armeniaca vulgaris var. ansu (Maxim.) T.T.Yu & L.T.Lu] (4.5), Gypsum Fibrosuum (7.5–10 g), Neolitsea cassia (L.) Kosterm. [Lauraceae; Cinnamomum cassia (L.) J.Presl] (4.5), Alisma plantago-aquatica subsp. orientale (Sam.) Sam. [Alismataceae; Alisma orientale (Sam.) Juz.] (4.5), Polyporus umbellatus (Pers.) Fries [Polyporaceae] (4.5), Atractylodes macrocephala Koidz. [Asteraceae; Atractylis macrocephala (Koidz.) Hand. -Mazz.] (4.5), Poria cocos (Schw.) Wolf [Polyporaceae] (7.5), Bupleurum chinense DC. [Apiaceae; Bupleurum octoradiatum Bunge] (8), Scutellaria baicalensis Georgi [Lamiaceae; Scutellaria macrantha Fisch. ex Rchb.] (3), Zingiber officinale Roscoe [Zingiberaceae; Amomum zingiber L.] (4.5), Pinellia ternata (Thunb.) Makino [Araceae; Arum ternatum Thunb.] (4.5), Aster tataricus L.f. [Asteraceae; Aster bracteatus Turcz. ex Herder] (4.5), Tussilago farfara L. [Asteraceae; Cineraria farfara (L.)] (4.5), Iris domestica (L.) Goldblatt & Mabb. [Iridaceae; Belamcanda chinensis (L.) Redouté] (4.5), Asarum heterotropoides F.Schmidt [Aristolochiaceae; Asarum heterotropoides var. mandshuricum (Maxim.) Kitag.] (3), Dioscorea oppositifolia L. [Dioscoreaceae; Dioscorea opposita Thunb.] (6), Citrus × aurantium L. [Rutaceae; Aurantium × sinense (L.) Mill.] (3), Citrus × aurantium f. deliciosa (Ten.) M.Hiroe [Rutaceae; Citrus × reticulata Blanco] (3), Agastache rugosa (Fisch. & C.A.Mey.) Kuntze [Lamiaceae; Agastache formosana (Hayata) Hayata ex Makino & Nemoto] (4.5).
				Fuzhengyiqing prescription: Panax ginseng C.A.Mey. [Araliaceae; Aralia ginseng (C.A.Mey.) Baill.] (5), Astragalus mongholicus Bunge [Fabaceae; Astragali R.] (5), Dioscorea oppositifolia L. [Dioscoreaceae; Dioscorea opposita Thunb.] (10), Citrus × aurantium f. deliciosa (Ten.) M.Hiroe [Rutaceae; Citrus × reticulata Blanco] (5), Poria cocos (Schw.) Wolf [Polyporaceae] (15), *Lonicera japonica* Thunb. [Caprifoliaceae; Caprifolium japonicum (Thunb.) Dum.Cours.] (5), Mentha canadensis L. [Lamiaceae; Mentha arvensis var. glabrata (Benth.)] (3), Morus alba L. [Moraceae; Morus alba var. arabica Bureau] (5), Phragmites australis subsp. australis [Poaceae; Arundo phragmites L.] (15), Agastache rugosa (Fisch. & C.A.Mey.) Kuntze [Lamiaceae; Agastache formosana (Hayata) Hayata ex Makino & Nemoto] (5), Glycyrrhiza uralensis Fisch. ex DC. [Fabaceae; Glycyrrhiza asperrima var. uralensis (Fisch. ex DC.) Regel & Herder] (3), *Nelumbo nucifera* Gaertn. [Nelumbonaceae; Nelumbium indicum Poir.] (3).
Liu et al. (2023)	Qiangshen Kangyi Decoction (granules)	Sichuan Neo-Green Pharmaceutical Technology Development Co., Ltd.	Partially reported^a^	Astragalus mongholicus Bunge [Fabaceae; Astragali R.] (15), *Lonicera japonica* Thunb. [Caprifoliaceae; *Caprifolium japonicum* (Thunb.) Dum.Cours.] (9), Atractylodes lancea (Thunb.) DC. [Asteraceae; *Atractylis chinensis* (Bunge) DC.] (5), Agastache rugosa (Fisch. & C.A.Mey.) Kuntze [Lamiaceae; *Agastache formosana* (Hayata) Hayata ex Makino & Nemoto] (6), Reynoutria japonica Houtt. [Polygonaceae; *Fallopia japonica *(Houtt.) Ronse Decr.] (6), Mentha canadensis L. [Lamiaceae; *Mentha arvensis* var. *glabrata* (Benth.)] (6).
Xie et al. (2023)	Yiqi Kangfei decoction (decoction)	NR	Partially reported^b^	Codonopsis pilosula (Franch.) Nannf. [Campanulaceae; *Campanumoea pilosula* Franch.] (6), Atractylodes macrocephala Koidz. [Asteraceae; *Atractylis macrocephala* (Koidz.) Hand. -Mazz.] (5), Atractylodes lancea (Thunb.) DC. [Asteraceae; *Atractylis chinensis* (Bunge) DC.] (5), Poria cocos (Schw)Wolf [Polyporaceae] (5), Citrus × aurantium f. deliciosa (Ten.) M.Hiroe [Rutaceae; *Citrus × reticulata* Blanco] (5), Agastache rugosa (Fisch. & C.A.Mey.) Kuntze [Lamiaceae; *Agastache formosana* (Hayata) Hayata ex Makino & Nemoto] (5), Wurfbainia villosa (Lour.) Škorničk. & A.D.Poulsen [Zingiberaceae; *Amomum villosum* Lour.] (3),Trichosanthes kirilowii Maxim. [Cucurbitaceae; *Anguina kirilowii *(Maxim.) Kuntze] (10), Stemona japonica (Blume) Miq. [Stemonaceae; *Roxburghia japonica *Blume] (10), Astragalus mongholicus Bunge [Fabaceae; Astragali R.] (15), Saposhnikovia divaricata (Turcz. ex Ledeb.) Schischk. [Apiaceae; *Ledebouriella seseloides* (Hoffm.) H.Wolff] (5), Prunus armeniaca var. armeniaca [Rosaceae; *Armeniaca vulgaris* var. *ansu* (Maxim.) T.T.Yu & L.T.Lu] (3), Curcuma longa L. [Zingiberaceae; *Stissera curcuma *Giseke] (5), Pinellia ternata (Thunb.) Makino [Araceae; *Arum ternatum* Thunb.] (10).
Zhen. 2021	CHM (Moxibustion: Artemisia argyi Lévl. et Van (Aiye).)	—	—	—

NR: not reported. CHM: Chinese herbal medicine. No quality control reports and chemical analysis report for the botanical drugs were provided in any of the studies.

^a^ Extraction temperature and time and amount of the provoked extract were reported but not the amount of the initial solvent.

^b^ Only the amount of provoked extract was reported.

^c^ Referred to simply as “boiling (decoction) or dissolved with boiled water (granules)”.

^d^ The additional botanical drugs based on symptoms were in the form of granules.

### 3.4 Characteristics and quality assessments

Among the RCTs, seven described the random sequence generation method (Liu et al., 2020b; Wang F et al., 2021; Xiao et al., 2021; Yan et al., 2021; Zheng, 2021; Liu et al., 2023) and five mentioned the randomization method (Wang YL et al., 2021; Wang ZZ et al., 2021; Yang et al., 2021; Zhang, 2021; Xie et al., 2023) and two studies reported the implementation of allocation concealment (Liu et al., 2020a; Liu et al., 2020b). Six studies reported the loss to follow-up cases (Wang et al., 2020; Wang F et al., 2021; Wang YL et al., 2021; Yan et al., 2021; Zheng and Lu, 2021; Liu et al., 2023). Seven studies had scores lower than four on the modified Jadad scale, indicating poor overall quality of research. The average NOS score for observational studies was 7.4 (Fang et al., 2020; Lv et al., 2020; Wang et al., 2020; Zheng and Lu, 2021; Wang et al., 2022), suggesting the high quality of the included studies. The summary of the included studies in the analysis were shown in Table 4 and Table 5.

**TABLE 4:** Modified Jadad scale for the included RCTs.

Author (year)	Generation of randomization	Randomization allocation	Blinding	Dropouts and withdrawals	Modified	quality
	allocation sequence (0–2 points)	concealment (0–2 points)	(0–2 points)	(0–1 point)	Jadad scale	
Yan et al. (2021)	2	1	0	1	4	High
Wang ZZ et al. (2021)	1	0	0	0	1	Low
Zhang. (2021)	1	0	0	0	1	Low
Wang YL et al. (2021)	1	0	0	1	2	Low
Zheng. (2021)	2	1	0	0	3	Low
Xiao et al. (2021)	2	1	0	0	3	Low
Liu et al. (2020a)	2	2	0	0	4	High
Liu et al. (2020b)	2	2	0	0	4	High
Wang F et al. (2021)	2	1	0	1	4	High
Yang et al. (2021)	1	0	0	0	1	Low
Liu et al. (2023)	2	1	0	1	4	High
Xie et al. (2023)	1	0	0	0	1	Low

**TABLE 5:** Newcastle-Ottawa risk of bias assessment.

	Selection				Comparability		outcome			Score	Risk of bias
	The exposed	The non-exposed	Ascertainment of exposure	outcome was not present in the study		Assessment of outcome		Was follow-up long enough for the outcome to occur	Complete accounting		
Lv et al., (2020)	1	1	1	1	2	1		0	0	7	Low
Fang et al., (2020)	1	1	1	1	2	1		0	0	7	Low
Wang et al., (2022)	1	1	1	1	2	1		0	0	7	Low
Zheng and Lu. (2021)	1	1	1	1	2	1		0	0	7	Low
Wang et al., (2020)	1	1	1	1	2	1		1	1	9	Low

### 3.5 Primary outcomes

#### 3.5.1 COVID-19 incidence

**FIGURE 2:**
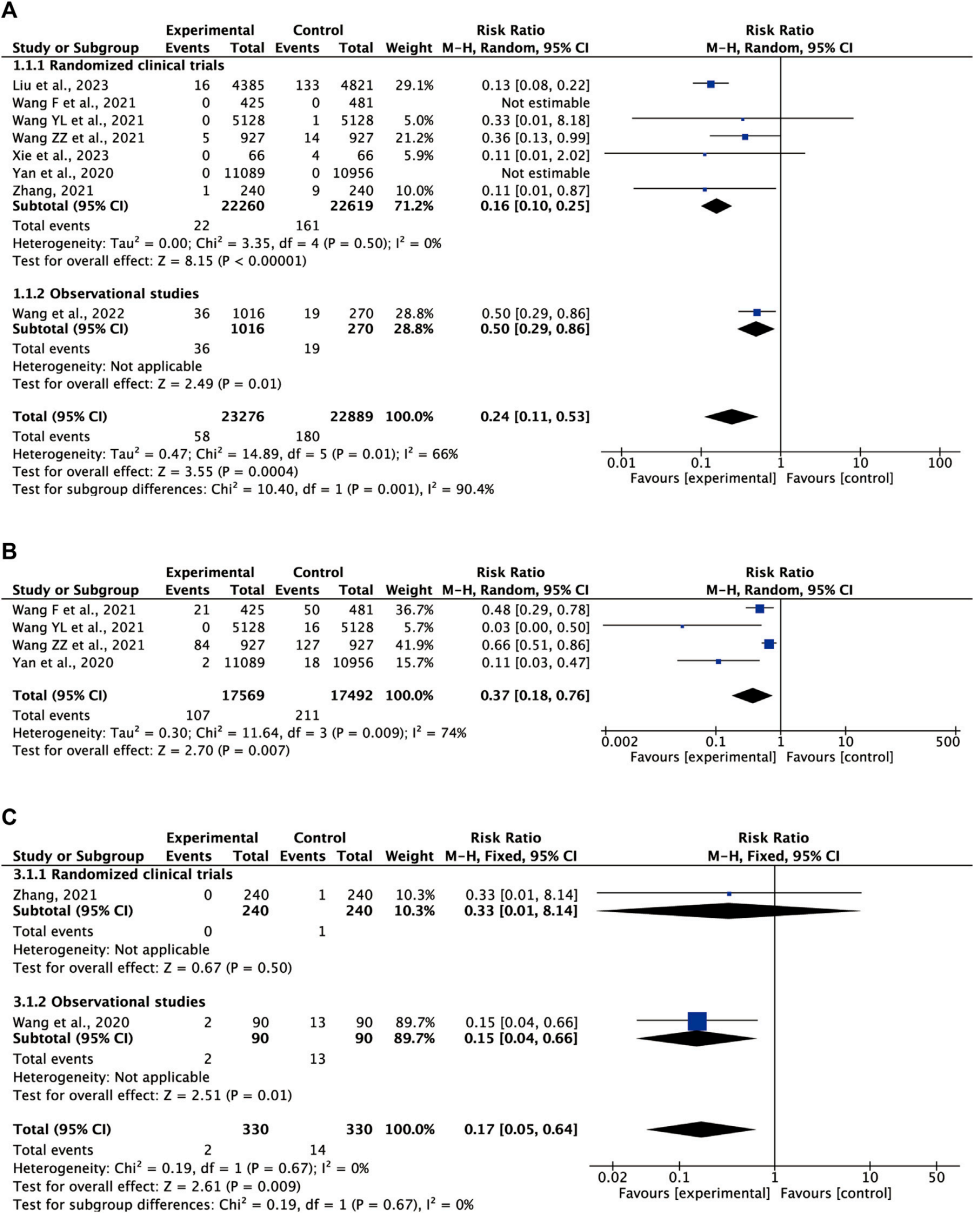
Meta-analysis results of CHM on primary outcomes **(A)** COVID-19 incidence **(B)** influenza incidence **(C)** severe pneumonia incidence.

The incidence of COVID-19 was investigated in eight clinical trials involving 46,165 patients (Wang et al., 2022; Wang F et al., 2021; Wang YL et al., 2021; Wang ZZ et al., 2021; Yan et al., 2021; Zhang, 2021; Xie et al., 2023; Liu et al., 2023). Compared to the non-treatment or conventional control group, the use of oral CHM resulted in a substantially reduced incidence of COVID-19 onset (RR = 0.24, 95% CI = 0.11–0.53, *p* = 0.0004). The studies exhibited significant heterogeneity (*I* ^*2*^ = 66%, *p* = 0.01); necessitating a random-effects model (Figure 2A).

#### 3.5.2 Influenza incidence

Given the similarity in symptoms between COVID-19 and influenza, distinguishing them based solely on symptoms during the early changes of the infection is difficult. The susceptible population or suspected cases of COVID-19 are also likely to develop into influenza, so the incidence of influenza was investigated. A total of four studies assessed the influenza incidence, including 35,061 participants who were susceptible to COVID-19, with 17,569 individuals in the CHM group and 17,492 individuals in the control group (Wang F et al., 2021; Wang YL et al., 2021; Wang ZZ et al., 2021; Yan et al., 2021). The result based on a random-effects model (*I* ^*2*^ = 74%, *p* = 0.009) indicated that oral CHM could reduce the incidence of influenza (RR = 0.37, 95% CI = 0.18–0.76) (Figure 2B).

#### 3.5.3 Severe pneumonia incidence

Two trials including 660 individuals provided data on the incidence of severe pneumonia (Wang et al., 2020; Zhang, 2021). The heterogeneity across trials was zero (*I* ^*2*^ = 0%, *p* = 0.67); thus, a fixed-effects model was performed (Figure 2C). The pooled data showed that oral CHM could reduce the incidence of severe pneumonia (RR = 0.17, 95% CI = 0.05–0.64, *p* = 0.009).

### 3.6 Secondary outcomes

#### 3.6.1 Immunological parameters

##### 3.6.1.1 IgA

**FIGURE 3:**
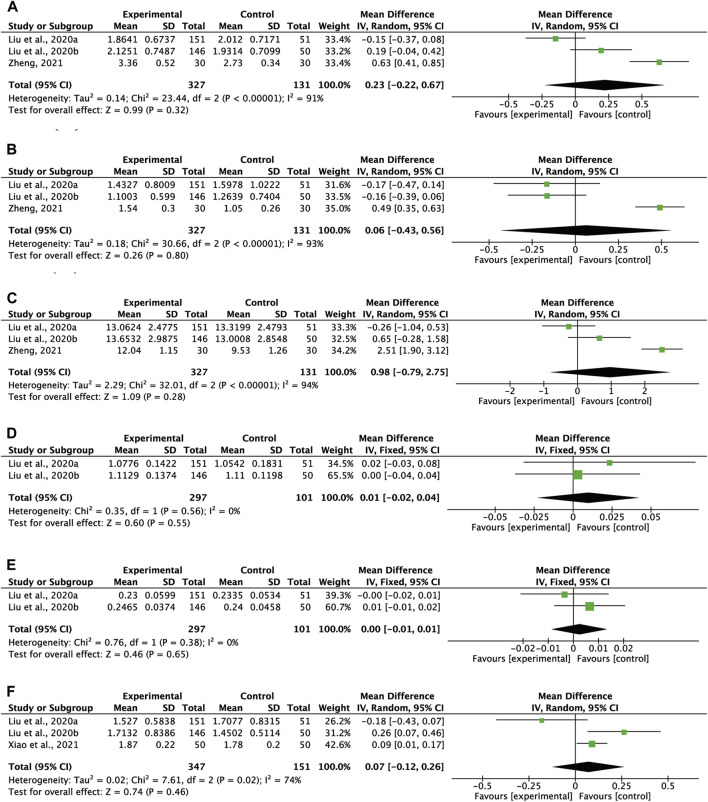
Meta-analysis results of immune system based on **(A)** IgA **(B)** IgM **(C)** IgG **(D)** C3 **(E)** C4 **(F)** CD4^+^/CD8^+^.

Three RCTs investigated the impact of CHM on the IgA ratio (Liu et al., 2020a; Liu et al., 2020b; Zheng, 2021), with 327 participants in the CHM group and 131 participants in the no-treatment group. In Zheng’s study, the CHM treatment group received external therapy (moxibustion), while in Liu’s studies, the CHM group received oral botanical drugs. The data from Liu’s studies (Liu et al., 2020a; Liu et al., 2020b) had a non-normal distribution. We calculated the sample mean and difference according to the original data’s median and interquartile range (Wan et al., 2014; Luo et al., 2018). The pooled data did not show any significant improvement in the IgA ratio in the CHM group (WMD = 0.23, 95% CI = -0.22-0.67, *p* = 0.32) based on the random-effects model, used due to the presence of significant heterogeneity across trials (*I* ^*2*^ = 91%, *p* < 0.00001) (Figure 3A).

##### 3.6.1.2 IgM

The effect of CHM on IgM was investigated in three RCTs (Liu et al., 2020a; Liu et al., 2020b; Zheng, 2021). Liu’s research (Liu et al., 2020a; Liu et al., 2020b) presented non-normally distributed data. Following previous studies (Wan et al., 2014; Luo et al., 2018), we calculated the sample median and sample differences using the provided data. The CHM therapy did not result in a statistically significant increase in IgM levels (WMD = 0.06, 95% CI = −0.43–0.56, *p* = 0.80). Since there was a high level of heterogeneity across trials (*I* ^*2*^ = 93%, *p* < 0.00001), we used a random-effects model (Figure 3B).

##### 3.6.1.3 IgG

Three RCTs provided data on the IgG level comparing the CHM group with a blank control group (Liu et al., 2020a; Liu et al., 2020b; Zheng, 2021). Notably, the effect of CHM on IgG was controversial as an increase in IgG levels was reported in two studies (Liu et al., 2020a; Liu et al., 2020b) while a decrease in IgG levels was presented in one (Zheng, 2021). A random-effects model was selected due to the significant heterogeneity across trials (*I* ^*2*^ = 94%, *p* < 0.00001) (Figure 3C). The pooled analysis did not reveal a significant difference in IgG between the CHM group and the blank control group (WMD = 0.98, 95% CI = −0.79–2.75, *p* = 0.28).

##### 3.6.1.4 C3

The impact of CHM on C3 was reported in two RCTs (Liu et al., 2020a; Liu et al., 2020b). No significant difference was found between the oral CHM group and the blank control group (WMD = 0.01, 95% CI = −0.02–0.04, *p* = 0.55). A fixed-effects model was employed given the absence of heterogeneity. (Figure 3D).

##### 3.6.1.5 C4

Two RCTs investigated the impact of oral CHM on C4 level, reporting a decreasing trend in C4 levels in both the CHM group and the non-intervention group, with no significant difference between the groups (Liu et al., 2020a; Liu et al., 2020b) (WMD = 0.00, 95% CI = −0.01–0.01, *p* = 0.65). Due to the absence of heterogeneity, a fixed-effects model was employed (Figure 3E).

##### 3.6.1.6 CD4^+^/CD8^+^

Three RCTs examined the impact of oral CHM used alone (Liu et al., 2020a; Liu et al., 2020b) or as an adjuvant (Xiao et al., 2021) on the CD4^+^/CD8^+^ ratio. Xiao’s study suggested a significant increase in the CD4+/CD8+ ratio in the CHM treatment group could significantly increase than in the conventional treatment group (Xiao et al., 2021). However, Liu’s studies indicated that there was no significant difference in CD4^+^/CD8^+^ between the CHM treatment group and the blank control group. Pooled analysis showed no significant on CD4^+^/CD8^+^ (WMD = 0.07, 95% CI = −0.12–0.26, *p* = 0.46, Figure 3F). A random-effects model was used, as heterogeneity was high (*I* ^*2*^ = 74%, *p* = 0.02).

#### 3.6.2 Disappearance rate of symptoms

##### 3.6.2.1 Disappearance rate of fever

**FIGURE 4:**
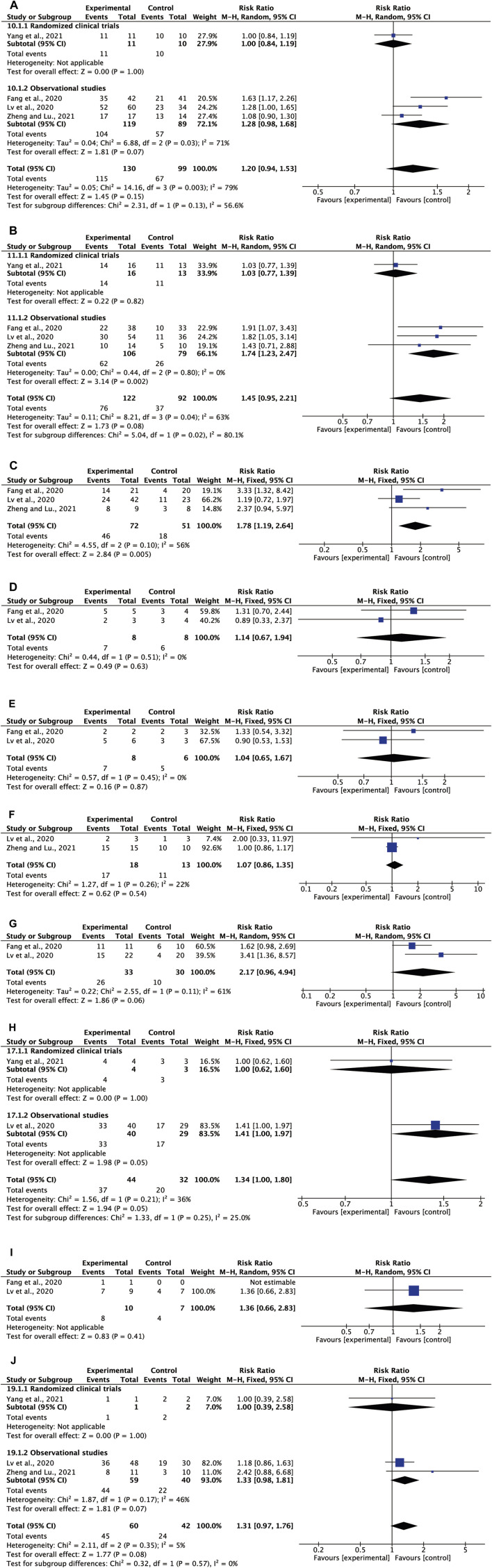
(Continued).

One RCT (Yang et al., 2021) and three observational studies (Fang et al., 2020; Lv et al., 2020; Zheng and Lu, 2021) assessed the efficacy of oral CHM as an adjuvant on the disappearance rate of fever, including 130 patients in the CHM group and 99 patients in the control group. The random-effects model was used due to the significant heterogeneity among the trials (*I* ^*2*^ = 79%, *p* = 0.003). No significant difference was observed between the oral CHM group and conventional treatment group on the disappearance of fever (RR = 1.20, 95% CI = 0.94–1.53, *p* = 0.15, Figure 4A), with similar results for subgroup analysis based on study design.

##### 3.6.2.2 Disappearance rate of cough

Figure 4B showed the effect of oral CHM as an adjuvant on the disappearance rate of cough. The random-effects model was used since the *I* ^*2*^ value was 63%. The meta-analysis pooling 214 patients did not indicate a significant difference between the oral CHM treatment group and the conventional treatment group on cough disappearance (RR = 1.45, 95% CI = 0.95–2.21, *p* = 0.08) (Fang et al., 2020; Lv et al., 2020; Yang et al., 2021; Zheng and Lu, 2021). The sensitivity analysis (Table 6) suggested that Yang’s study (Yang et al., 2021) was the probable source of heterogeneity since the *I* ^*2*^ value became zero when this research was excluded from the analysis (RR = 1.74, 95% CI = 1.23-2.47, *p* = 0.002).

**TABLE 6:** Summarized data for sensitivity analysis.

Outcome	Study	Data with study removed RR/WMD (95% CI)	*I* ^*2*^ (%)	Test for overall effect Z P	
COVID-19 incidence	Liu et al. (2023)	0.42 [0.27, 0.66]	0%	3.77	0.0002
	Wang F et al. (2021)	0.24 [0.11, 0.53]	66%	3.55	0.0004
	Wang YL et al. (2021)	0.24 [0.10, 0.55]	73%	3.37	0.0007
	Wang YL et al. (2021)	0.22 [0.08, 0.57]	72%	3.07	0.002
	Xie et al. (2023)	0.26 [0.11, 0.59]	73%	3.22	0.001
	Yan et al., 2021	0.24 [0.11, 0.53]	66%	3.55	0.0004
	Zhang (2021)	0.27 [0.11, 0.62]	72%	3.06	0.002
	Wang et al. (2022)	0.16 [0.10, 0.25]	0%	8.15	<0.00001
Influenza incidence	Wang F et al. (2021)	0.18 [0.03, 1.16]	82%	1.80	0.07
	Wang YL et al. (2021)	0.46 [0.26, 0.84]	70%	2.54	0.01
	Wang YL et al. (2021)	0.17 [0.03, 0.83]	74%	2.19	0.03
	Yan et al., 2021	0.50 [0.28, 0.92]	67%	2.22	0.03
Severe pneumonia incidence	Zhang (2021)	0.15 [0.04, 0.66]	Not applicable	2.51	0.01
	Wang et al. (2020)	0.33 [0.01, 8.14]	Not applicable	0.67	0.50
IgA	Liu et al. (2020b)	0.41 [-0.01, 0.84]	86%	1.89	0.06
		0.24 [-0.52, 1.00]	96%	0.62	0.54
	Zheng (2021)	0.02 [-0.31, 0.36]	77%	0.13	0.90
IgM	Liu et al. (2020b)	0.17 [-0.47, 0.81]	96%	0.52	0.60
		0.18 [-0.46, 0.82]	93%	0.54	0.59
	Zheng (2021)	−0.16 [-0.35, 0.02]	0%	1.76	0.08
IgG	Liu et al. (2020b)	1.62 [-0.20, 3.43]	91%	1.74	0.08
		1.14 [-1.57, 3.85]	97%	0.82	0.41
	Zheng (2021)	0.16 [-0.73, 1.05]	53%	0.36	0.72
C3	Liu et al. (2020b)	0.00 [-0.04, 0.04]	Not applicable	0.14	0.89
		0.02 [-0.03, 0.08]	Not applicable	0.83	0.41
C4	Liu et al. (2020b)	0.01 [-0.01, 0.02]	Not applicable	0.91	0.37
		−0.00 [-0.02, 0.01]	Not applicable	0.39	0.69
CD4^+^/CD8^+^	Liu et al. (2020b)	0.15 [-0.01, 0.32]	61%	1.84	0.07
		−0.02 [-0.28, 0.24]	76%	0.15	0.88
	Xiao et al. (2021)	0.05 [-0.39, 0.48]	87%	0.21	0.83
Disappearance rate of fever	Yang et al. (2021)	1.28 [0.98, 1.68]	71%	1.81	0.07
	Zheng and Lu. (2021)	1.26 [0.88, 1.80]	84%	1.25	0.21
	Fang et al. (2020)	1.10 [0.92, 1.30]	54%	1.06	0.29
	Lv et al. (2020)	1.18 [0.84, 1.67]	87%	0.96	0.34
Disappearance rate of cough	Yang et al. (2021)	1.74 [1.23, 2.47]	0%	3.14	0.002
	Zheng and Lu. (2021)	1.48 [0.85, 2.59]	77%	1.37	0.17
	Fang et al. (2020)	1.34 [0.83, 2.15]	64%	1.19	0.23
	Lv et al. (2020)	1.35 [0.82, 2.24]	65%	1.18	0.24
Disappearance rate of sputum	Zheng and Lu. (2021)	1.86 [0.67, 5.17]	74%	1.18	0.24
	Fang et al. (2020)	1.50 [0.80, 2.83]	39%	1.26	0.21
	Lv et al. (2020)	2.81 [1.46, 5.41]	0%	3.09	0.002
Disappearance rate of nasal obstruction	Fang et al. (2020)	0.89 [0.33, 2.37]	Not applicable	0.24	0.81
	Lv et al. (2020)	1.31 [0.70, 2.44]	Not applicable	0.85	0.40
Disappearance rate of runny nose	Fang et al. (2020)	0.90 [0.53, 1.53]	Not applicable	0.39	0.69
	Lv et al. (2020)	1.33 [0.54, 3.32]	Not applicable	0.62	0.54
Disappearance rate of sore throat	Zheng and Lu. (2021)	2.00 [0.33, 11.97]	Not applicable	0.76	0.45
	Lv et al. (2020)	1.00 [0.86, 1.17]	Not applicable	0.00	1.00
Disappearance rate of shortness of breath	Fang et al. (2020)	3.41 [1.36, 8.57]	Not applicable	2.61	0.009
	Lv et al. (2020)	1.62 [0.98, 2.69]	Not applicable	1.87	0.06
Disappearance rate of fatigue	Yang et al. (2021)	1.41 [1.00, 1.97]	Not applicable	1.98	0.05
	Lv et al. (2020)	1.00 [0.62, 1.60]	Not applicable	0.00	1.00
Disappearance rate of muscle pain	Fang et al. (2020)	1.36 [0.66, 2.83]	Not applicable	0.83	0.41
	Lv et al. (2020)	Not estimable	Not applicable	Not applicable	Not applicable
Disappearance rate of poor appetite	Yang et al. (2021)	1.33 [0.98, 1.81]	46%	1.81	0.07
	Zheng and Lu. (2021)	1.17 [0.86, 1.58]	0%	1.02	0.31
	Lv et al. (2020)	1.87 [0.88, 3.99]	48%	1.62	0.11
Adverse reactions	Liu et al. (2023)	9.82 [0.04, 2649.58]	95%	0.80	0.42
	Wang YL et al. (2021)	21.04 [0.02, 18510.59]	96%	0.88	0.38
	Xie et al. (2023)	23.33 [0.16, 3324.05]	95%	1.24	0.21
	Yan et al. (2021)	12.79 [0.02, 7407.94]	96%	0.79	0.43
	Zhang (2021)	23.33 [0.16, 3324.05]	95%	1.24	0.21
	Lv et al. (2020)	122.20 [24.43, 611.35]	0%	5.85	<0.00001

##### 3.6.2.3 Disappearance rate of sputum

The efficacy of oral CHM as an adjuvant compared with conventional treatment on sputum disappearance was evaluated in three studies with 123 participants (Fang et al., 2020; Lv et al., 2020; Zheng and Lu, 2021). The *I* ^*2*^ value was 56%, thus a random-effects model was used. The study showed that the sputum disappearance rate was not significantly improved when compared to the conventional control group (RR = 1.78, 95% CI = 1.19–2.64, *p* = 0.005, Figure 4C). After further sensitivity analysis, we found that the heterogeneity was caused by Lv’s study (Lv et al., 2020) dropping to zero when removed from the equation (Table 6).

##### 3.6.2.4 Disappearance rate of nasal obstruction

Figure 4D depicted the impact of oral CHM on the disappearance rate of nasal obstruction. There were eight cases in the group that received CHM as an adjuvant, and eight in the control group (Fang et al., 2020; Lv et al., 2020). No significant improvement in the disappearance rate of nasal congestion was found between the oral CHM group and the conventional treatment group (RR = 1.14, 95% CI = 0.67–1.94, *p* = 0.63). We opted for a model with fixed effects due to the absence of heterogeneity (*I* ^*2*^ value of 0%).

##### 3.6.2.5 Disappearance rate of runny nose

Two trials, including 14 participants, investigated the disappearance rate of runny nose between the oral CHM group and control group (Fang et al., 2020; Lv et al., 2020). Given the absence of heterogeneity, a fixed-effects model was used (*I* ^*2*^ = 0%, *p* = 0.45). The meta-analysis did not show a significant improvement in the runny nose disappearance rate when compared with the conventional treatment group (RR = 1.04, 95% CI = 0.65–1.67, *p* = 0.87, Figure 4E).

##### 3.6.2.6 Disappearance rate of sore throat

Two trials, including 31 participants, evaluated the efficacy of oral CHM as an adjuvant for sore throat (Lv et al., 2020; Zheng and Lu, 2021). The fixed-effects model was used given the low heterogeneity (*I* ^*2*^ = 22%, *p* = 0.26). No significant improvement was found in the disappearance rate of sore throat between the oral CHM group and the conventional treatment group (RR = 1.07, 95% CI = 0.86–1.35, *p* = 0.54, Figure 4F).

##### 3.6.2.7 Disappearance rate of shortness of breath

Figure 4G illustrated the efficacy of oral CHM as an adjuvant on the disappearance rate of shortness of breath, involving 33 patients in the oral CHM group and 30 patients in the control group (Fang et al., 2020; Lv et al., 2020). The random-effects model was applied as the *I* ^*2*^ was 61% (*p* = 0.11). The analysis showed that the disappearance rate was not significantly improved in the oral CHM group compared to the conventional treatment group (RR = 2.17, 95% CI = 0.96–4.94, *p* = 0.06).

##### 3.6.2.8 Disappearance rate of fatigue

The impact of oral CHM as an adjuvant on the disappearance rate of fatigue was measured in two trials with a total of 76 patients (Lv et al., 2020; Yang et al., 2021). There were 44 individuals in the CHM group and 32 individuals in the control group. The fixed-effects model was utilized to analyze the data given the homogeneity (*I* ^*2*^ = 36%, *p* = 0.21) with no significant difference observed in the disappearance rate of fatigue between the CHM group and the conventional treatment group (RR = 1.34, 95% CI = 1.00–1.80, *p* = 0.05, Figure 4H).

##### 3.6.2.9 Disappearance rate of muscle pain

Figure 4I showed the effect of oral CHM as an adjuvant on the disappearance rate of muscle pain. The pooled analysis included two trials with 17 patients (Fang et al., 2020; Lv et al., 2020). Due to the insignificance in heterogeneity, we applied a random-effects model with findings indicating that the disappearance rate of muscle pain was not significantly improved in the oral CHM group (RR = 1.36, 95% CI = 0.66–2.83, *p* = 0.41).

##### 3.6.2.10 Disappearance rate of poor appetite

Three trials involving a total of 102 individuals investigated the disappearance rate of poor appetite (Lv et al., 2020; Yang et al., 2021; Zheng and Lu, 2021). The fixed-effects model was employed since heterogeneity was 5% (*p* = 0.35). The meta-analysis did not show a significant improvement in the disappearance rate of poor appetite in the oral CHM group compared to the conventional treatment group (RR = 1.31, 95% CI = 0.97–1.76, *p* = 0.08, Figure 4J).

### 3.7 Adverse reactions

**FIGURE 5:**
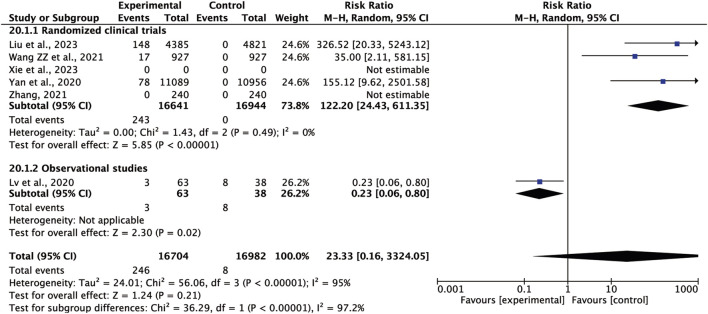
Meta-analysis results of adverse reaction.

Six studies identified the presence of adverse reactions with the most common adverse reactions being symptoms related to the gastrointestinal system (Lv et al., 2020; Wang ZZ et al., 2021; Yan et al., 2021; Zhang, 2021; Liu et al., 2023). The *I* ^*2*^ value was 95% (*p* < 0.00001), thus the random-effects model was used. Oral CHM as an adjuvant therapy did not increase the risk of adverse reactions when compared to the conventional treatment alone. In fact, a tendency towards a reduction in the risk of adverse reactions was exhibited (RR = 23.33, 95% CI = 0.16–3324.05, *p* = 0.21) (Figure 5). After further sensitivity analysis (Table 6), we identified Lv’s study (Lv et al., 2020) as the source of heterogeneity, since the *I* ^*2*^ value dropped to zero when this study was excluded from the meta-analysis.

### 3.8 Publication bias

A funnel plot could not be generated to assess publication bias because there were less than 10 studies included in the meta-analysis for each outcome.

### 3.9 Certainty of the evidence

The quality of evidence for each outcome indicator was assessed using GRADEpro. Notably, the outcome quality of the COVID-19 incidence in RCTs, C3, and C4 had a moderate quality of evidence. In contrast, the quality of findings relevant to the incidence of influenza and severe pneumonia in RCTs, and CD4^+^/CD8^+^ in RCTs was deemed low. Other outcomes (IgA, IgM, IgG, disappearance rate of symptoms and adverse reactions) had a very low quality of evidence. A detailed evaluation of the evidence was shown in the Supplementary Material.

## 4 Discussion

### 4.1 Overview

The rapid worldwide spread of COVID-19 has impacted more people in a shorter period of time than any other recorded illness in history. As a result, the world has been facing one of the worst public health emergencies of international concern in recent years. Amid this crisis, COVID-19 remains imperfectly understood, and existing prevention and treatment strategies fall short of addressing clinical demands.

Notably, the development of a vaccine has provided hope for people worldwide. Some researchers believe the virus will be eliminated *via* herd immunity if enough individuals acquire immunity (Frederiksen et al., 2020; Randolph and Barreiro, 2020). However, challenges arose due to the virus’s rapid evolution and the lengthy vaccine development timelines. Immunity to COVID-19 in the general population particularly in vulnerable groups characterized by age, co-morbidities, or compromised immunity before infection or highly effective vaccination was limited. (Stringhini et al., 2020; Leong et al., 2021). Individuals in these categories are especially susceptible if they interact with high-risk communities.

Currently, there is no longer a mandatory requirement for nucleic acid testing in many regions of the world. When individuals have suspected symptoms or a recent history of high-risk exposure, many Western medical interventions are deemed unsuitable for use in the absence of a confirmed diagnosis. It is therefore of great significance to utilize the potential of CHM to enhance the body’s immune system and prevent the occurrence of diseases. For individuals who have already been infected, a subsequent infection increases the risk of adverse health consequences. Measures need to be taken to reduce the possibility of reinfection (Boufidou et al., 2023). Additionally, due to inadequate medical facilities in some areas coupled with differences in cultural customs and beliefs, preventive measures such as vaccines for COVID-19 cannot be universally applied (Onyeaghala et al., 2023). Therefore, given the current research challenge, the focus of the global medical community is enhancing the population’s capacity to prevent illnesses.

Numerous studies highlight the efficacy of CHM. This ancient and effective medicine has helped save lives by slowing the spread of COVID-19 (Huang et al., 2021; Zhao et al., 2021). Thus, to better support the efficacy of CHM in preventing COVID-19, this study rigorously examined and summarized the most robust data available to analyze CHM’s effectiveness in the prevention of COVID-19.

Our comprehensive meta-analysis encompassing 17 studies investigated the efficacy of CHM in preventing COVID-19. Our results indicated that the oral CHM used as an adjuvant did not significantly improve patient symptoms compared to the conventional treatment alone. Similarly, when comparing the use of CHM alone to the blank control group, there was no significant impact on patients’ immune function. However, our findings demonstrated that CHM combination treatment might decrease the prevalence of influenza and severe pneumonia in the general population. Both COVID-19 and influenza are respiratory diseases with a certain incubation period and overlapping symptoms, making early-stage differentiation based solely on symptoms difficult. Furthermore, the susceptible population or suspected cases of COVID-19 are also likely to develop into influenza. As such, our study incorporated influenza incidence as one of the primary outcomes.

Our research indicated that CHM could boost immunity and prevent both COVID-19 and influenza. This was associated with the effect of CHM on the generation of immune cells and cytokines. Maintaining a healthy state relies on the functionality of the immunological regulatory system, which is responsible for maintaining internal environmental stability and resilience against external. Complement C3 and C4 play crucial roles as integral constituents of the innate immune system. The activation of the complement system has the potential to exacerbate the infection caused by COVID-19 *via* the induction of a cytokine storm (Polycarpou et al., 2020). Biomarkers of excessive activation and consumption of the complement system can also be used to predict the prognosis in patients (Sinkovits et al., 2021). The inhibition of the C3 and C4 pathways has been shown to effectively impede the complement system and serve as a potential therapeutic approach for managing COVID-19 (Pires et al., 2023). According to our meta-analysis, C3 and C4 levels in the intervention group receiving CHM treatment tended to decrease. It is possible that during the epidemic, a significant portion of the population exhibited hyper-immunity due to various factors, such as exposure to specific natural or social environments and psychological influences. CHM could possibly serve to temper this immunological hyperactive response and thus foster more balanced immunological functions. Participants in this research were comprised of individuals suspected of having COVID-19, members from high-risk demographics (such as children and the elderly), and healthy individuals. Compared with the control group, the clinical symptoms of fever and cough improved when CHM was applied promptly, according to the findings of a retrospective survey of suspected COVID-19 patients (Fang et al., 2020). In addition, the probability of transitioning from mild to severe COVID-19 and of new coronavirus infection was reduced, presenting a crucial point in preventing the progression of the illness with the potential for disease reversal.

Within immunocompromised and high-risk older populations, there was a trend towards an increase in the CD4^+^/CD8^+^ ratio, implying that CHM might play a role in enhancing patients’ cellular immunity. Moreover, our analysis unveiled that the preventative effect of CHM against COVID-19 was more pronounced in children and older adults (Fang et al., 2020; Wang et al., 2022). We believe that the following factors contribute to this observation. First, older adults have more underlying health conditions, and the prescriptions used in the trial contained several botanical drugs that have the effect of tonifying qi, improving immunity, and addressing underlying diseases, thus, better achieving the effect of preventing COVID-19. Recent research also indicates that the bioactive components of Lianhua Qingwen granules target cytokines and participate in the NFκB and JAK/STAT signaling pathways, which are involved in the mouldability of the host immunity against viruses (Chen et al., 2023). Compared to young and middle-aged people, a relatively small proportion of older adults have received vaccinations, emphasizing the preventive effect of herbal medicine. Second, according to Traditional Chinese Medicine (TCM) theory, children are considered delicate in terms of their zang-fu organs and immature physique. Nonetheless, their unique vitality renders them susceptible to pathogenic factors but their recovery tends to be more rapid than other age groups (Wang et al., 2007). Consequently, intervention in the early stage of the disease can effectively stop the progress of the disease or even prevent the disease before it occurs.

### 4.2 Mechanisms associated with WE medicine

The use of WE medicine, specifically in the form of CHM as an adjuvant, illuminates the compatibility of Eastern and Western medicine. These two modalities, present significant cultural differences. While Eastern medicine emphasizes the laws of motion and interrelationships at the macroscopic level of matter, Western medicine is more concerned with the fineness of matter at the microscopic level. Despite these differences, both systems prioritize preserving life, and their complementary attributes can be combined to facilitate further advancements in medical research and practice. The studies analyzed in this meta-analysis used a diverse range of CHM formats, including Chinese patent medications, herbal formula granules, and CHM. It is important to note that this CHM can be categorized into two unique groups, each with unique qualities and traits. The first category comprises Astragalus mongholicus Bunge [Fabaceae; Astragali R.], Saposhnikovia divaricata (Turcz. ex Ledeb.) Schischk. [Apiaceae; Ledebouriella seseloides (Hoffm.) H. Wolff] and Atractylodes macrocephala Koidz. [Asteraceae; Atractylis macrocephala (Koidz.) Hand. -Mazz.], whereas the second category consists of *L. japonica* Thunb. [Caprifoliaceae; Caprifolium japonicum (Thunb.) Dum. Cours.], Glycyrrhiza uralensis Fisch. ex DC. [Fabaceae; Glycyrrhiza asperrima var. uralensis (Fisch. ex DC.) Regel & Herder], Platycodon grandiflorus (Jacq.) A. DC. [Campanulaceae; Campanula glauca Thunb.], Rhodiola rosea L. [Crassulaceae; Rhodiola sachalinensis Boriss.], and Forsythia suspensa (Thunb.) Vahl [Oleaceae; Syringa suspensa Thunb.]. The first group comprised the traditional and classic formula known as the Yupingfeng Powder. This formula can be traced back to the “Danxi Xinfa,“written during the Yuan Dynasty. Whereas Astragalus mongholicus Bunge [Fabaceae; Astragali R.] is the root of the leguminous plant Astragalus in Mongolia (Chinese Pharmacopoeia Commission Volume, 2020). Recent pharmacological investigations have revealed that Astragalus can increase immunity (Cho and Leung, 2007), reduce inflammation, protect cells from free radical damage, and modulate immune functions (Qi et al., 2017). Several studies shed light on the primary therapeutic effect of Yupingfeng Powder and its effectiveness in improving the barrier function of the airway mucosa. This is accomplished by restoring and safeguarding the airway mucosa’s natural outcomes and controlling the body’s immunological function (Gao et al., 2009; Wang et al., 2022; Yu et al., 2022). Contemporary pharmacology has demonstrated that Forsythia suspensa (Thunb.) Vahl [Oleacea; Syringa suspensa Thunb.] has antipyretic, anti-inflammatory, and antiviral effects (Wang et al., 2018). Agastache rugosa (Fisch. & C.A.Mey.) Kuntze [Lamiaceae; Agastache formosana (Hayata) Hayata ex Makino & Nemoto] has antifungal, anti-acute lung injury, immunomodulatory, anti-inflammatory, anti-tumor, and anti-inflammatory effects (Zhuang et al., 2020). *Lonicera japonica* Thunb. [Caprifoliaceae; Caprifolium japonicum (Thunb.) Dum. Cours.] has broad-spectrum anti-pathogenic microorganisms, antiviral, antipyretic, anti-inflammatory actions, and other effects (Li et al., 2021). Rhodiola rosea L. [Crassulaceae; Rhodiola sachalinensis Boriss.] has mainly antiviral, antioxidant, and anti-acute lung damage effects (Chiang et al., 2015; Xu et al., 2018).

“Preventive therapy” is a characteristical concept in TCM. It suggests that intervening at the earliest possible stages of illness development can effectively halt the disease progression or, at the very least, significantly decelerate it (Qiu et al., 2020). In contrast, the prevalence rate may increase without early intervention. According to the principles of TCM, COVID-19 falls under the category of “epidemic illnesses”. The emergence of every infectious illness is connected with two facets: “righteousness” and “evil”. The goal of “preventive therapy theory” is to encourage righteousness and enhance physical fitness to prevent individuals from being invaded by harmful materials. Presently, researchers are addressing the dangers posed by COVID-19. Timely, whole-process and systematic methods are necessary, all while benefiting from the potential therapeutic effects of CHM. Indeed, the preventative impact of CHM on the new coronary pneumonia opens up avenues for future research in preventing influenza and other respiratory infectious disorders.

## 5 Differences from previous meta-analyses of CHM for COVID-19

Our systematic review stands out on several fronts. Firstly, while the previously published systematic reviews of CHM for COVID-19 have focused on the aspect of treatment of confirmed cases, this review focused on the prevention of COVID-19 including undiagnosed and suspected cases. To our knowledge, this review is the first of its kind to analyze the efficacy of CHM in preventing COVID-19.

Secondly, in the context of epidemics involving infectious diseases such as COVID-19 and influenza, the balance of the immune system serves as a critical determinant in the body’s ability to resist infection and interrupt disease progression. Compared with other published reviews, more indicators of the immune system were incorporated as secondary outcomes of this review to evaluate the role of CHM in regulating immunity.

Thirdly, we have taken a measured and objective approach to present both favorable and less favorable findings relating to the role of CHM in pandemic management. The results of our analysis support the preventive potential of CHM concerning the occurrence of COVID-19, influenza, and severe pneumonia, however, without a significant effect on improving indicators of the immune system. Additionally and in contrast with previous studies, no significant improvement in clinical symptoms with CHM was found.

Lastly, this systematic review illuminated the potential of WE medicine. In the face of unmet medical demands, the integration of Western and Eastern medical paradigms offers a promising avenue. This review underscores the significance of such an integrative approach in addressing current healthcare challenges effectively.

## 6 Limitations

Several limitations to this study should be acknowledged. Foremost, the majority of RCT studies did not discuss allocation concealment, and only a few studies addressed withdrawal and loss to follow-up rates, both of which have the potential to introduce biases into the study’s execution and measurements. Additionally, all the included cohort studies adopted a retrospective design. While they contributed valuable insights, this retrospective approach might have affected the comparability between study groups. Furthermore, there were no clinical trials related to the use of CHM in regions beyond China, thus limiting the generalizability and the applicability of the findings. Inconsistencies within the age distribution, disease history, and treatment course of the included patients, were a source of heterogeneity, and further complication comparability. Moreover, the numbers of involved patients in some subgroup analyses were limited, especially in the evaluation of symptom improvement, underscoring the need for future research.

## 7 Conclusion

The findings of this meta-analysis revealed beneficial effects of oral CHM, whether administered alone or used as an adjuvant treatment, in reducing the disease development rate and in preventing COVID-19 infections compared to blank control or conventional treatment alone. Nevertheless, in light of the limited number and inferior quality of the included publications, further studies are required. We recommend conducting centralized studies with large samples, utilizing randomized, controlled, and blinded designs to strengthen the evidence base.

## Data Availability

The original contributions presented in the study are included in the article/Supplementary Material, further inquiries can be directed to the corresponding authors.

## Abbreviations

CHM, Chinese herbal medicine; COVID-19, Coronavirus disease 2019; WHO, World Health Organization; RR, Relative risk; CI, Confidence intervals; R0, Basic reproduction number; SARS, Severe acute respiratory syndrome; SARS-CoV-2, severe acute respiratory syndrome coronavirus 2; RNA, Ribonucleic acid, RCTs, Randomized controlled trials; NOS, Newcastle-Ottawa score; OR, Odds ratios; WMD, Weighted mean difference; SMD, Standardized mean difference; TCM, Traditional Chinese Medicine.
