# Supplementary material for: An updated meta-analysis of Chinese herbal medicine for the prevention of COVID-19 based on Western-Eastern medicine
Source: Front Pharmacol. 2023 Nov 13;14:1257345. doi: 10.3389/fphar.2023.1257345 (PMC10693348; doi:10.3389/fphar.2023.1257345)
Supplement: Supplementary file 1 [file Table1.DOCX]

| **Certainty assessment** | | | | | | | **№ of patients** | | **Effect** | | **Certainty** | **Importance** |
| --- | --- | --- | --- | --- | --- | --- | --- | --- | --- | --- | --- | --- |
| **№ of studies** | **Study design** | **Risk of bias** | **Inconsistency** | **Indirectness** | **Imprecision** | **Other considerations** | **CHM** | **Control** | **Relative (95% CI)** | **Absolute (95% CI)** |  |  |
| **COVID-19 incidence** | | | | | | | | | | | | |
| 7 | randomised trials | serious^a^ | not serious | not serious | not serious | none | 22/22260 (0.1%) | 161/22619 (0.7%) | **RR 0.16** (0.10 to 0.25) | **6 fewer per 1,000** (from 6 fewer to 5 fewer) | ⨁⨁⨁◯ Moderate | CRITICAL |
| **COVID-19 incidence** | | | | | | | | | | | | |
| 1 | observational studies | not serious | not serious | not serious | not serious | none | 36/1016 (3.5%) | 19/270 (7.0%) | **RR 0.50** (0.29 to 0.86) | **35 fewer per 1,000** (from 50 fewer to 10 fewer) | ⨁⨁◯◯ Low | CRITICAL |
| **influenza incidence** | | | | | | | | | | | | |
| 4 | randomised trials | serious^a^ | serious^b^ | not serious | not serious | none | 107/17569 (0.6%) | 211/17492 (1.2%) | **RR 0.37** (0.18 to 0.76) | **8 fewer per 1,000** (from 10 fewer to 3 fewer) | ⨁⨁◯◯ Low | CRITICAL |
| **severe pneumonia incidence** | | | | | | | | | | | | |
| 1 | randomised trials | serious^a^ | not serious | not serious | serious^c^ | none | 0/240 (0.0%) | 1/240 (0.4%) | **RR 0.33** (0.01 to 8.14) | **3 fewer per 1,000** (from 4 fewer to 30 more) | ⨁⨁◯◯ Low | CRITICAL |
| **severe pneumonia incidence** | | | | | | | | | | | | |
| 1 | observational studies | not serious | not serious | not serious | not serious | none | 2/90 (2.2%) | 13/90 (14.4%) | **RR 0.15** (0.04 to 0.66) | **123 fewer per 1,000** (from 139 fewer to 49 fewer) | ⨁⨁◯◯ Low | CRITICAL |
| **IgA** | | | | | | | | | | | | |
| 3 | randomised trials | not serious | very serious^d^ | not serious | serious^c^ | none | 327 | 131 | - | MD **0.23 higher** (0.22 lower to 0.67 higher) | ⨁◯◯◯ Very low | CRITICAL |
| **IgM** | | | | | | | | | | | | |
| 3 | randomised trials | not serious | very serious^d^ | not serious | serious^c^ | none | 327 | 131 | - | MD **0.06 higher** (0.43 lower to 0.56 higher) | ⨁◯◯◯ Very low | CRITICAL |
| **IgG** | | | | | | | | | | | | |
| 3 | randomised trials | not serious | very serious^d^ | not serious | serious^c^ | none | 327 | 131 | - | MD **0.98 higher** (0.79 lower to 2.75 higher) | ⨁◯◯◯ Very low | CRITICAL |
| **C3** | | | | | | | | | | | | |
| 2 | randomised trials | not serious | not serious | not serious | serious^c^ | none | 297 | 101 | - | MD **0.01 higher** (0.02 lower to 0.04 higher) | ⨁⨁⨁◯ Moderate | CRITICAL |
| **C4** | | | | | | | | | | | | |
| 2 | randomised trials | not serious | not serious | not serious | serious^c^ | none | 297 | 101 | - | MD **0**  (0.01 lower to 0.01 higher) | ⨁⨁⨁◯ Moderate | CRITICAL |
| **CD4+/CD8+** | | | | | | | | | | | | |
| 3 | randomised trials | not serious | serious^b^ | not serious^b^ | serious^c^ | none | 347 | 151 | - | MD **0.07 higher** (0.12 lower to 0.26 higher) | ⨁⨁◯◯ Low | CRITICAL |
| **disappearance rate of fever** | | | | | | | | | | | | |
| 1 | randomised trials | serious^a^ | not serious | not serious | very serious^e^ | none | 11/11 (100.0%) | 10/10 (100.0%) | **RR 1.00** (0.84 to 1.19) | **0 fewer per 1,000** (from 160 fewer to 190 more) | ⨁◯◯◯ Very low | CRITICAL |
| **disappearance rate of fever** | | | | | | | | | | | | |
| 3 | observational studies | serious | serious^b^ | not serious | very serious^e^ | none | 104/119 (87.4%) | 57/89 (64.0%) | **RR 1.28** (0.98 to 1.68) | **179 more per 1,000** (from 13 fewer to 436 more) | ⨁◯◯◯ Very low | CRITICAL |
| **disappearance rate of cough** | | | | | | | | | | | | |
| 1 | randomised trials | serious^a^ | not serious | not serious | very serious^e^ | none | 14/16 (87.5%) | 11/13 (84.6%) | **RR 1.03** (0.77 to 1.39) | **25 more per 1,000** (from 195 fewer to 330 more) | ⨁◯◯◯ Very low | CRITICAL |
| **disappearance rate of cough** | | | | | | | | | | | | |
| 3 | observational studies | not serious | not serious | not serious | serious^c^ | none | 10/14 (71.4%) | 5/10 (50.0%) | **RR 1.45** (0.95 to 2.21) | **225 more per 1,000** (from 25 fewer to 605 more) | ⨁◯◯◯ Very low | CRITICAL |
| **disappearance rate of sputum** | | | | | | | | | | | | |
| 3 | observational studies | not serious | serious^b^ | not serious | very serious^e^ | none | 46/72 (63.9%) | 18/51 (35.3%) | **RR 1.78** (1.19 to 2.64) | **275 more per 1,000** (from 67 more to 579 more) | ⨁◯◯◯ Very low | CRITICAL |
| **disappearance rate of nasal obstruction** | | | | | | | | | | | | |
| 2 | observational studies | not serious | not serious | not serious | very serious^e^ | none | 7/8 (87.5%) | 6/8 (75.0%) | **RR 1.14** (0.67 to 1.94) | **105 more per 1,000** (from 247 fewer to 705 more) | ⨁◯◯◯ Very low | CRITICAL |
| **disappearance rate of runny nose** | | | | | | | | | | | | |
| 2 | observational studies | not serious | not serious | not serious | very serious^e^ | none | 7/8 (87.5%) | 5/6 (83.3%) | **RR 1.04** (0.65 to 1.67) | **33 more per 1,000** (from 292 fewer to 558 more) | ⨁◯◯◯ Very low | CRITICAL |
| **disappearance rate of sore throat** | | | | | | | | | | | | |
| 2 | observational studies | not serious | not serious | not serious | very serious^e^ | none | 17/18 (94.4%) | 11/13 (84.6%) | **RR 1.07** (0.86 to 1.35) | **59 more per 1,000** (from 118 fewer to 296 more) | ⨁◯◯◯ Very low | CRITICAL |
| **disappearance rate of shortness of breath** | | | | | | | | | | | | |
| 2 | observational studies | not serious | serious^b^ | not serious | very serious^e^ | none | 26/33 (78.8%) | 10/30 (33.3%) | **RR 2.17** (0.96 to 4.94) | **390 more per 1,000** (from 13 fewer to 1,000 more) | ⨁◯◯◯ Very low | CRITICAL |
| **disappearance rate of fatigue** | | | | | | | | | | | | |
| 1 | randomised trials | serious^a^ | not serious | not serious | very serious^e^ | none | 4/4 (100.0%) | 3/3 (100.0%) | **RR 1.00** (0.62 to 1.60) | **0 fewer per 1,000** (from 380 fewer to 600 more) | ⨁◯◯◯ Very low | CRITICAL |
| **disappearance rate of fatigue** | | | | | | | | | | | | |
| 1 | observational studies | not serious | not serious | not serious | very serious^e^ | none | 33/40 (82.5%) | 17/29 (58.6%) | **RR 1.41** (1.00 to 1.97) | **240 more per 1,000** (from 0 fewer to 569 more) | ⨁◯◯◯ Very low | CRITICAL |
| **disappearance rate of muscle pain** | | | | | | | | | | | | |
| 2 | observational studies | not serious | not serious | not serious | very serious^e^ | none | 8/10 (80.0%) | 4/7 (57.1%) | **RR 1.36** (0.66 to 2.83) | **206 more per 1,000** (from 194 fewer to 1,000 more) | ⨁◯◯◯ Very low | CRITICAL |
| **disappearance rate of poor appetite** | | | | | | | | | | | | |
| 1 | randomised trials | serious^a^ | not serious | not serious | very serious^e^ | none | 1/1 (100.0%) | 2/2 (100.0%) | **RR 1.00** (0.39 to 2.58) | **0 fewer per 1,000** (from 610 fewer to 1,000 more) | ⨁◯◯◯ Very low | CRITICAL |
| **disappearance rate of poor appetite** | | | | | | | | | | | | |
| 2 | observational studies | not serious | not serious | not serious | very serious^e^ | none | 44/59 (74.6%) | 22/40 (55.0%) | **RR 1.33** (0.98 to 1.81) | **182 more per 1,000** (from 11 fewer to 446 more) | ⨁◯◯◯ Very low | CRITICAL |
| **adverse reaction** | | | | | | | | | | | | |
| 5 | randomised trials | serious^a^ | not serious | not serious | serious^c^ | none | 243/16641 (1.5%) | 0/16944 (0.0%) | **RR 122.20** (24.43 to 611.35) | **0 fewer per 1,000** (from 0 fewer to 0 fewer) | ⨁⨁◯◯ Low | CRITICAL |
| **adverse reaction** | | | | | | | | | | | | |
| 1 | observational studies | not serious | not serious | not serious | serious^f^ | none | 3/63 (4.8%) | 8/38 (21.1%) | **RR 0.23** (0.06 to 0.80) | **162 fewer per 1,000** (from 198 fewer to 42 fewer) | ⨁◯◯◯ Very low | CRITICAL |

**CI:** confidence interval; **MD:** mean difference; **RR:** risk ratio

#### Explanations

a. Jadad scored an average of 3 points or less because of randomized allocation sequence, randomization allocation, or the blinding generated were not clear, we decided to downgrade the quality of evidence to the risk of bias.

b. There was great heterogeneity among the studies included in the analysis, with heterogeneity ranging from 50% to 75%. When these problems and inconsistencies were considered, we decided to go down one level.

c. The 95% confidence interval exceeded the equivalence line and we decided to downgrade the quality evidence of the study.

d. There was significant heterogeneity among the studies included in the analysis, with heterogeneity greater than or equal to 75%. When considering these problems and inconsistencies, we decided to take it to the next level.

e. The 95% confidence intervals exceeded the equivalence line and the total sample size of the included studies was less than 300. Overall, we decided to reduce the quality of the evidence for this study.

f. The total sample size of the included studies was less than 300. Overall, we decided to reduce the quality of the evidence for this study.
