# Supplementary material for: An updated meta-analysis of Chinese herbal medicine for the prevention of COVID-19 based on Western-Eastern medicine
Source: Front Pharmacol. 2023 Nov 13;14:1257345. doi: 10.3389/fphar.2023.1257345 (PMC10693348; doi:10.3389/fphar.2023.1257345)
Supplement: Supplementary file 2 [file Table2.DOCX]

**Search strategies**

| Literature databases | Search items | Items found |
| --- | --- | --- |
| (1) PubMed | TS=("COVID 19" OR "SARS-CoV-2 Infection" OR "Infection, SARS-CoV-2" OR "Infection, SARS-CoV-2" OR "SARS CoV 2 Infection" OR "SARS CoV 2 Infection" OR "SARS-CoV-2 Infections" OR "2019 Novel Coronavirus Disease" OR "2019 Novel Coronavirus Infection" OR "2019-nCoV Disease" OR "2019 nCoV Disease" OR "2019-nCoV Diseases" OR "Disease, 2019-nCoV" OR "COVID-19 Virus Infection" OR "COVID 19 Virus Infection" OR "COVID-19 Virus Infections" OR "Infection, COVID-19 Virus" OR "Virus Infection, COVID-19" OR "Coronavirus Disease 2019" OR "Disease 2019, Coronavirus" OR "Coronavirus Disease-19" OR "Coronavirus Disease 19" OR "Severe Acute Respiratory Syndrome Coronavirus 2 Infection" OR "SARS Coronavirus 2 Infection" OR "COVID-19 Virus Disease" OR "COVID 19 Virus Disease" OR "COVID-19 Virus Diseases" OR "Disease, COVID-19 Virus" OR "Virus Disease, COVID-19" OR "2019-nCoV Infection" OR "2019 nCoV Infection" OR "2019-nCoV Infections" OR "Infection, 2019-nCoV" OR "COVID19" OR "COVID-19 Pandemic" OR "COVID 19 Pandemic" OR "Pandemic, COVID-19" OR "COVID-19 Pandemics") AND TS=("Traditional Chinese Medicine" OR "Traditional Medicine" OR "Chinese; Hsueh" OR "Chung I" OR "Zhong Yi Xue" OR "Chinese Traditional Medicine" OR "Chung I Hsueh" OR "Chinese Medicine, Traditional" OR "Chinese herbal medicine" OR "Chinese Herbal Drugs" OR "Chinese Drugs" OR "Plant" OR "Chinese medicine formulae" OR "Chinese medicine formulations" OR "Chinese herb" OR "Chinese herb therapy" OR "Medicine, Traditional" OR "Medicine, East Asian Traditional" OR "Drugs, Chinese Herbal" OR "Herbal Drugs, Chinese" OR "herbal medicine" OR "herb remedy" OR "herb therapy") AND TS=("clinical trials") | 341 |
| (2)Science Direct | #1  Title, abstract or author-specified keywords: "COVID 19" OR "SARS-CoV-2 Infection" OR "Infection, SARS-CoV-2" OR "Infection, SARS-CoV-2" OR "SARS CoV 2 Infection" OR "SARS CoV 2 Infection" OR "SARS-CoV-2 Infections" OR "2019 Novel Coronavirus Disease"  #2  ("Traditional Chinese Medicine" OR "Traditional Medicine" OR "Chinese; Hsueh" OR "Chung I" OR "Zhong Yi Xue" OR "Chinese Traditional Medicine" OR "Chung I Hsueh" OR "Chinese Medicine, Traditional" ) AND ("clinical trials")  #1 AND #2 | 1680 |
| (3)Web of science | TS = ("COVID 19" OR "SARS-CoV-2 Infection" OR "Infection, SARS-CoV-2" OR "Infection, SARS-CoV-2" OR "SARS CoV 2 Infection" OR "SARS CoV 2 Infection" OR "SARS-CoV-2 Infections" OR "2019 Novel Coronavirus Disease" OR "2019 Novel Coronavirus Infection" OR "2019-nCoV Disease" OR "2019 nCoV Disease" OR "2019-nCoV Diseases" OR "Disease, 2019-nCoV" OR "COVID-19 Virus Infection" OR "COVID 19 Virus Infection" OR "COVID-19 Virus Infections" OR "Infection, COVID-19 Virus" OR "Virus Infection, COVID-19" OR "Coronavirus Disease 2019" OR "Disease 2019, Coronavirus" OR "Coronavirus Disease-19" OR "Coronavirus Disease 19" OR "Severe Acute Respiratory Syndrome Coronavirus 2 Infection" OR "SARS Coronavirus 2 Infection" OR "COVID-19 Virus Disease" OR "COVID 19 Virus Disease" OR "COVID-19 Virus Diseases" OR "Disease, COVID-19 Virus" OR "Virus Disease, COVID-19" OR "2019-nCoV Infection" OR "2019 nCoV Infection" OR "2019-nCoV Infections" OR "Infection, 2019-nCoV" OR "COVID19" OR "COVID-19 Pandemic" OR "COVID 19 Pandemic" OR "Pandemic, COVID-19" OR "COVID-19 Pandemics") AND TS=("Traditional Chinese Medicine" OR "Traditional Medicine" OR "Chinese; Hsueh" OR "Chung I" OR "Zhong Yi Xue" OR "Chinese Traditional Medicine" OR "Chung I Hsueh" OR "Chinese Medicine, Traditional" OR "Chinese herbal medicine" OR "Chinese Herbal Drugs" OR "Chinese Drugs" OR "Plant" OR "Chinese medicine formulae" OR "Chinese medicine formulations" OR "Chinese herb" OR "Chinese herb therapy" OR "Medicine, Traditional" OR "Medicine, East Asian Traditional" OR "Drugs, Chinese Herbal" OR "Herbal Drugs, Chinese" OR "herbal medicine" OR "herb remedy" OR "herb therapy") AND TS = ("clinical trials")  Timespan: All years. Indexes: SCI-EXPANDED. | 408 |
| (4)Google Scholar | ("COVID 19" OR "SARS-CoV-2 Infection" OR "Infection, SARS-CoV-2" OR "Infection, SARS-CoV-2" OR "SARS CoV 2 Infection" OR "SARS CoV 2 Infection" OR "SARS-CoV-2 Infections" OR "2019 Novel Coronavirus Disease" OR "2019 Novel Coronavirus Infection" OR "2019-nCoV Disease" OR "2019 nCoV Disease" OR "2019-nCoV Diseases" OR "Disease, 2019-nCoV" OR "COVID-19 Virus Infection" OR "COVID 19 Virus Infection") AND ("COVID19" OR "COVID-19 Pandemic" OR "COVID 19 Pandemic" OR "Pandemic, COVID-19" OR "COVID-19 Pandemics" AND ALL=("Traditional Chinese Medicine" OR "Traditional Medicine" OR "Chinese; Hsueh" OR "Chung I" OR "Zhong Yi Xue" OR "Chinese Traditional Medicine" OR "Chung I Hsueh" OR "Chinese Medicine, Traditional" OR "Chinese herbal medicine" OR "Chinese Herbal Drugs") AND ("clinical trials") | 4540 |
| (5)Embase | ('covid 19'/exp OR 'sars-cov-2 infection'/exp OR 'infection, sars-cov-2' OR 'sars cov 2 infection'/exp OR 'sars-cov-2 infections' OR '2019 novel coronavirus disease'/exp OR '2019 novel coronavirus infection'/exp OR '2019-ncov disease'/exp OR '2019 ncov disease'/exp OR '2019-ncov diseases' OR 'disease, 2019-ncov' OR 'covid-19 virus infection' OR 'covid 19 virus infection' OR 'covid-19 virus infections' OR 'infection, covid-19 virus' OR 'virus infection, covid-19' OR 'coronavirus disease 2019'/exp OR 'disease 2019, coronavirus' OR 'coronavirus disease-19'/exp OR 'coronavirus disease 19'/exp OR 'severe acute respiratory syndrome coronavirus 2 infection'/exp OR 'sars coronavirus 2 infection'/exp OR 'covid-19 virus disease' OR 'covid 19 virus disease' OR 'covid-19 virus diseases' OR 'disease, covid-19 virus' OR 'virus disease, covid-19' OR '2019-ncov infection'/exp OR '2019 ncov infection'/exp OR '2019-ncov infections' OR 'infection, 2019-ncov' OR 'covid19'/exp OR 'covid-19 pandemic' OR 'covid 19 pandemic' OR 'pandemic, covid-19' OR 'covid-19 pandemics') AND ('traditional chinese medicine'/exp OR 'traditional medicine'/exp OR 'chinese; hsueh' OR 'chung i' OR 'zhong yi xue' OR 'chinese traditional medicine'/exp OR 'chung i hsueh' OR 'chinese medicine, traditional' OR 'chinese herbal medicine'/exp OR 'chinese herbal drugs' OR 'chinese drugs' OR 'plant'/exp OR 'chinese medicine formulae' OR 'chinese medicine formulations' OR 'chinese herb'/exp OR 'chinese herb therapy' OR 'medicine, traditional'/exp OR 'medicine, east asian traditional'/exp OR 'drugs, chinese herbal'/exp OR 'herbal drugs, chinese' OR 'herbal medicine'/exp OR 'herb remedy' OR 'herb therapy') AND 'clinical trials' | 390 |
| (6)the Cochrane Library | ("COVID 19" OR "SARS-CoV-2 Infection" OR "Infection, SARS-CoV-2" OR "Infection, SARS-CoV-2" OR "SARS CoV 2 Infection" OR "SARS CoV 2 Infection" OR "SARS-CoV-2 Infections") AND ("Traditional Chinese Medicine" OR "Traditional Medicine" OR "Chinese; Hsueh" OR "Chung I" OR "Zhong Yi Xue" OR “Chinese herbal medicine”) AND ("clinical trials") | 170 |
| (7)China National Knowledge Infrastructure Database | #1  (主题=“新型冠状病毒肺炎”) OR (主题=“新冠肺炎”) OR (主题="COVID-19") OR (主题=“2019 冠状病毒病”) OR (主题=“冠状病毒肺炎”) OR (主题=“疑似人群”)  #2  (主题=“预防”) OR (主题=“治未病”) OR (主题=“防控”)OR (主题=“治疗”)  #3  (篇关摘=“中成药”) OR (篇关摘=“草药”) OR (篇关摘=“中草药”) OR (篇关摘=“中药”) OR (篇关摘=“中医药”) OR (篇关摘=“传统医学”) OR (篇关摘=“补充疗法”) OR (篇关摘=“替代疗法”)  #4  (全文=”临床”) OR (全文=”对照”) OR (全文=”随机”) OR (全文=”观察”)  #1 AND #2 AND #3 AND #4 | 1582 |
| (8)Wan Fang database Search strategy | 主题=("新型冠状病毒" or "新冠" or "COVID-19" or "2019冠状病毒病" or "冠状病毒") AND 主题=("预防" or "治未病" or "治疗" or "防控") AND 主题=("中药" or "草药" or "中草药" or "传统医药" or "中医药" or "替代疗法" or "补充疗法")) AND ("临床" or "对照" or "随机" or "观察") | 1614 |
| (9)Chongqing VIP Chinese Science and Technology Periodical Database (VIP) | ((((M="新型冠状病毒" OR "新冠") OR M="COVID-19") OR M="2019 冠状病毒病") OR M="冠状病毒") AND (((M="预防" OR "治未病") OR M="治疗") OR M="防控") AND ((((((M="中药" OR "草药") OR M="中草药") OR M="传统医药") OR M="中医药") OR M="替代疗法") OR M="补充疗法") AND (((U="临床" OR U="对照") OR U="随机") OR U="观察") | 418 |
| (10) SinoMed | [( ""新型冠状病毒""[常用字段:智能] OR ""COVID19""[常用字段:智能] OR ""新冠"OR"g冠状病毒""[常用字段:智能]) AND( ""预防""[常用字段:智能] OR ""治未病""[常用字段:智能] OR ""治疗""[常用字段:智能] OR ""防控""[常用字段:智能]) AND( ""临床""[常用字段:智能] OR "“观察""[常用字段:智能] OR ""随机""[常用字段:智能] OR ""对照""[常用字段:智能](javascript:this.top.vpn_inject_scripts_window(this);vpn_eval((function%20()%20%7b%20toDoRelimitSearch();%20%7d).toString().slice(14,%20-2)))] | 369 |
| (11) WHO COVID-19 website | COVID-19 | 0 |
| Overall | / | 11512 |
